# Mining hidden knowledge: embedding models of cause–effect relationships curated from the biomedical literature

**DOI:** 10.1093/bioadv/vbac022

**Published:** 2022-04-07

**Authors:** Andreas Krämer, Jeff Green, Jean-Noël Billaud, Nicoleta Andreea Pasare, Martin Jones, Stuart Tugendreich

**Affiliations:** QIAGEN Digital Insights, Redwood City, CA 94063, USA

## Abstract

**Motivation:**

We explore the use of literature-curated signed causal gene expression and gene–function relationships to construct unsupervised embeddings of genes, biological functions and diseases. Our goal is to prioritize and predict activating and inhibiting functional associations of genes and to discover hidden relationships between functions. As an application, we are particularly interested in the automatic construction of networks that capture relevant biology in a given disease context.

**Results:**

We evaluated several unsupervised gene embedding models leveraging literature-curated signed causal gene expression findings. Using linear regression, we show that, based on these gene embeddings, gene–function relationships can be predicted with about 95% precision for the highest scoring genes. Function embedding vectors, derived from parameters of the linear regression model, allow inference of relationships between different functions or diseases. We show for several diseases that gene and function embeddings can be used to recover key drivers of pathogenesis, as well as underlying cellular and physiological processes. These results are presented as disease-centric networks of genes and functions. To illustrate the applicability of our approach to other machine learning tasks, we also computed embeddings for drug molecules, which were then tested using a simple neural network to predict drug–disease associations.

**Availability and implementation:**

Python implementations of the gene and function embedding algorithms operating on a subset of our literature-curated content as well as other code used for this paper are made available as part of the Supplementary data.

**Supplementary information:**

Supplementary data are available at *Bioinformatics Advances* online.

## 1 Introduction

Many experimental observations reported in the biomedical literature represent cause–effect relationships. Examples are observations that directly or indirectly couple the activation or inhibition of genes to the downstream regulation of other genes, or the activation or inhibition of biological functions. Collectively, such literature-derived causal relationships (Krämer *et al.*, 2014) can be viewed as the defining features of genes and functions, and therefore be exploited in machine learning (ML) models.

A widely used approach is the construction of mappings to high-dimensional vector representations (Hinton, 1986), so-called embeddings, that are at the heart of many modern ML methods. The most famous example for this is arguably the word2vec algorithm (Mikolov *et al.*, 2013), which uses word proximity in a text to encode semantic relationships in high-dimensional word embeddings. Embeddings have also been applied to graphs (Grover and Leskovec, 2016; Nelson *et al.*, 2019) and used in scientific contexts, for instance to discover latent knowledge in materials science (Tshitoyan *et al.*, 2019). In the biological context, embeddings for genes have been constructed from protein sequences (Yang *et al.*, 2018), protein–protein interaction networks (Cho *et al.*, 2016), coexpression data (Du *et al.*, 2019) and using text mining (Liang *et al.*, 2021; Xing *et al.*, 2018).

In this work, we explore the use of literature-curated signed causal gene expression and gene–function relationships to construct unsupervised embeddings of genes and functions. In contrast to protein–protein interactions or correlation measures like coexpression, causal gene expression relationships capture information about the behavior of a biological system as a whole in response to perturbations. Here, we make explicit use of the fact that causal interactions carry a sign which distinguishes between activating and inhibiting effects.

The obtained gene embeddings can be used to predict and prioritize genes affecting functions and diseases. We distinguish our approach from existing function prediction methods that aim to annotate previously uncharacterized genes with their predicted function, based on some form of ‘guilt-by-association’, i.e. the assumption that colocalized and interacting genes or proteins are more likely to be functionally correlated (Chen *et al.*, 2021). Here, in contrast, we are interested in the identification of the most relevant genes causally affecting a given function or disease. These genes can either be previously known to be associated with that function or purely predicted. In the context of diseases, gene prioritization approaches were previously developed based on matrix factorization (Natarajan and Dhillon, 2014; Zakeri *et al.*, 2018), but those do not distinguish between activating and inhibiting effects. In addition to gene embeddings, we also construct function embedding vectors that allow to infer previously unknown signed function–function relationships, including disease–function associations that point to disease mechanisms involving specific cell types or tissues.

Our embeddings are generally useful to construct biological networks that highlight some mechanism or key contexts. A recent example is the ‘Coronavirus Network Explorer’ (Krämer *et al.*, 2021), which uses an early version of our gene–function prediction approach to compute networks that connect severe acute respiratory syndrome coronavirus 2 (SARS-CoV-2) viral proteins to host cell functions. In the current paper, we illustrate the application to biological networks by constructing disease networks, which capture disease-underlying functions and associated key genes. Embeddings are not limited to genes, but can also be extended to other molecules including drugs. Such embedding feature vectors can then be used in other ML models trained for arbitrary prediction tasks. As an example, we demonstrate this for the prediction of drug–disease associations.

## 2 Methods

### 2.1 Literature-curated content

We employ the QIAGEN Knowledge Base (QKB), a structured collection of biomedical content that includes findings manually curated from the literature as well as content from third-party databases (https://digitalinsights.qiagen.com/products-overview/qiagen-knowledge-base/). The QKB was used to create a large-scale knowledge graph with nodes representing genes, chemical compounds, drugs, microRNAs, biological functions and diseases; and edges categorized into different edge types representing a variety of interactions such as gene expression, activation/inhibition, phosphorylation and protein–protein binding among others. For more details regarding QKB content, see Supplementary data, Section 1.

In this work, we particularly focus on two kinds of edges: (i) gene expression relationships that represent the causal effect of genes on the expression of other genes and (ii) causal gene–function and gene–disease edges that represent causal effects of genes on biological functions and diseases.

Here, causality relating to an edge A→B between two entities *A* and *B* is defined in the following way: There exists at least one published experimental observation, in some experimental context, that a change in some property of *A* (usually its activation, inhibition, over-expression, knockout, etc.) results in (i.e. ‘causes’) a measured response of *B*, e.g. its expression up- or downregulation if *B* is a gene, or its activation or inhibition (promotion/suppression) if *B* is a biological function or disease. Examples of these kind of edges, and their underlying literature findings are shown in Supplementary data, Section 1.2.

We only consider *signed* edges that have an associated direction of effect which is either activation (leading to an increase, sign: +1) or inhibition (leading to a decrease, sign: –1). All edges generally bundle a number of underlying literature findings from various experimental contexts, therefore edge signs reflect a consensus among all those contexts. Note that our approach explicitly excludes protein–protein binding edges since those do not represent causal effects and also do not carry an edge sign which is required by our method.

As part of an ontology, functions are organized in a hierarchy where, except for very general terms, parents inherit causal gene associations (and edge signs) from their descendants. In total, 6757 genes and 29 553 functions are included in our embedding model. Here and in the following, the term ‘function’ generally refers to both functions and diseases, unless we explicitly make the distinction.

### 2.2 Unsupervised gene embeddings

**Fig. 1.**
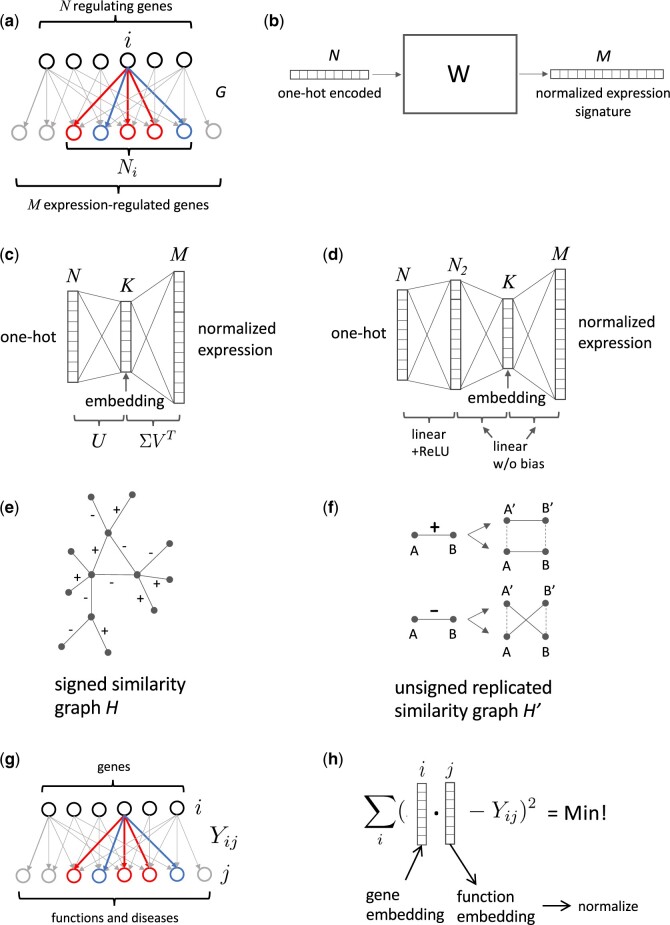
Gene and function embedding methods. (**a**) In the bipartite graph *G*, regulating genes are connected to expression-regulated genes by signed edges that represent upregulating and downregulating causal expression findings from the literature (for illustration, different colors represent different regulation directions). Embedding vectors are computed for the *N* regulating genes. *G* defines the signed, weighted adjacency matrix *W*. (**b**) *W* can be viewed as taking *N*-dimensional one-hot encoded gene vectors as input and outputting normalized *M*-dimensional vectors corresponding to the up/downregulation pattern. (**c**) The spectral method E1 uses a low-rank approximation $\tilde{W}=U\Sigma V^{T}$ to compute embedding vectors, which is equivalent to training a simple three-layer linear neural network without bias terms and MSE loss. (**d**) The neural network-based embedding strategy E2 extends the linear model by adding another layer which includes bias and an ReLU activation function. (**e**) The graph-based approach E3 uses a signed similarity graph *H* connecting similar and anti-similar genes. (**f**) From *H*, an unsigned graph *H*’ is constructed with a replicated set of nodes. *H*’ allows the computation of embeddings using the node2vec algorithm (Grover and Leskovec, 2016). (**g**) The gene–function (and disease) bipartite graph defines the bi-adjacency matrix *Y_ij_*. Genes are connected to functions and diseases by signed edges that represent activating and inhibiting causal effect findings from the literature. (**h**) Function (and disease) embedding vectors are computed as parameter vectors in a linear regression problem

In the following, we describe three approaches to derive unsupervised embeddings of genes from their downstream expression signatures defined by literature-curated signed causal gene expression relationships. The starting point is a bipartite graph *G* (see Fig. 1a) in which *N* genes (for which we will compute embeddings) are connected to their *M* expression-regulated target genes by signed edges that represent causal expression findings from the literature. From *G* we define the signed, weighted *N *×* M* bi-adjacency matrix *W*, $W_{ij}=\frac{s_{ij}}{\sqrt{N_{i}}}$, where $s_{ij}\in \{-1,0,1\}$ (activation: + 1, inhibition: –1, no edge: 0) and $N_{i}=\sum_{j}|s_{ij}|$ is the total number of genes that are regulated by gene *i*. The matrix *W* can be viewed as taking *N*-dimensional one-hot encoded gene vectors as input and outputting normalized *M*-dimensional vectors corresponding to the up/downregulation pattern (see Fig. 1b). Two of our embedding strategies (E1 and E2) are based on an approximation of the matrix *W*, which is associated with the compression of the one-hot encoded input into a lower-dimensional embedding space.

The ‘spectral’ embedding E1 uses a low-rank approximation of *W* based on singular value decomposition (Markovsky, 2012),

(1) $$\tilde{W}=U\Sigma V^{T},$$

where columns of the *N *×* K* matrix *U* are eigenvectors of the positive definite matrix $S=WW^{T}$, corresponding to its top *K* eigenvalues. Entries of the matrix *S* represent a signed ‘similarity’ of genes based on their downstream regulation patterns. Note that the normalization factor $1/\sqrt{N_{i}}$ used in the construction of *W* was chosen such that diagonal elements of *S* are equal to one, regardless of the number of regulated genes. The square roots of the eigenvalues of *S* form the matrix elements of the diagonal *K *×* K* matrix Σ, and *V* is an *M *×* K* matrix. One can think of *U* as projecting one-hot encoded vectors representing single genes onto *K*-dimensional embedding vectors, i.e. these embedding vectors are the rows of *U*, where $U^{T}U=I$. This spectral method of computing embedding vectors is equivalent (up to constant scale factors on embedding vector components) to training a simple three-layer linear neural network without bias terms and mean-squared error (MSE) loss (corresponding to the Frobenius norm of $\tilde{W}$), where embeddings are retrieved from the middle layer (Bermeitinger *et al.*, 2019; see Fig. 1c). The neural network-based embedding strategy E2 extends this linear model by adding another layer that includes bias and has a rectified linear unit (ReLU) activation function in order to capture non-linear effects (see Fig. 1d). Since there is no bias term between the final layers for both the E1 and E2 approaches, inverting the sign of an embedding vector will result in exactly the opposite effect on downstream-regulated genes.

For the third embedding strategy (E3), instead of using the signed similarity matrix *S*, we construct a signed similarity graph *H* that has a signed edge between two gene nodes *i* and *k* if the two genes exhibit a similar downstream regulation pattern. In particular, we compute the ‘z-score’ $z_{ik}=\frac{1}{\sqrt{N_{ik}}}\sum_{j}s_{ij}s_{kj}$ where $N_{ik}=\sum_{j}|s_{ij}||s_{kj}|$ is the number of coregulated genes and requires the absolute value of *z_ik_* to meet a certain cutoff for an edge to be present. The sign of an edge is given by the sign of *z_ij_* (see Fig. 1e). From *H*, we construct an *unsigned* graph *H*’ by replicating each node of *H* and connecting the replicated nodes in *H*’ either parallel (positive edge sign) or crosswise (negative edge sign) with unsigned edges as shown in Figure 1f. This construction of an unsigned graph *H*’ preserves the information contained in the edge signs of *H*. In the next step, we apply the node2vec graph embedding algorithm (Grover and Leskovec, 2016) that samples random walks in order to map the graph embedding problem to word2vec using the skip-gram approach (Mikolov *et al.*, 2013). Embedding vectors *u_i_* and *v_i_* are computed for all nodes in *H*’, where *u* and *v* denote the two replicas, one of which is used for the final gene embedding vectors.

### 2.3 Function embeddings

Functions are characterized by their causally associated genes that were curated from literature along with the respective direction of the effect (activation or inhibition). We construct function embedding vectors *p* in the same vector space as gene embedding vectors *x* such that their scalar product p·x approximates the effect of *x* on *p* (activation: p·x>0, inhibition: p·x<0, no effect: p·x≈0). This construction is in line with the symmetry described above: a gene with opposite causal expression signature, i.e. with the embedding vector -x has also the opposite effect −p·x on the function *p*.

Function embedding vectors are determined as follows: Let the matrix $Y=\{Y_{ij}\}$ represent the effect of gene *i* on a function *j* (activation: *Y_ij_* = 1, inhibition: $Y_{ij}=-1$, no effect: *Y_ij_* = 0) as curated from the literature (see Fig. 1g), then the embedding vector *p_j_* for each function *j* is determined independently by standard linear regression (using MSE loss; see Fig. 1h), i.e. by minimizing $\sum_{i}{\left(x_{i}\cdot p_{j}-Y_{ij}\right)}^{2}$. This leads to

(2) $$p_{j}={\left(U^{T}U\right)}^{-1}U^{T}y_{j},$$

where the matrix *U* has *K*-dimensional gene embedding vectors as rows, *y_j_* is a column vector of *Y* and it is assumed that the r.h.s. of Equation (2) is well-behaved, and no further regularization is needed, which is usually the case if K≪N. For the spectral method E1 in particular we have $U^{T}U=I$, which simplifies Equation (2) to $p_{j}=U^{T}y_{j}$. Note, that gene–function prediction is viewed as a regression problem, not classification, since the values of *Y_ij_* are ordered in a sequence, −1, 0, 1 and there could in principle be a continuous transition from ‘inhibition’, to ‘no effect’ to ‘activation’. We finalize the construction of function embedding vectors by also performing a normalization step, ${\tilde{p}}_{j}=\frac{p_{j}}{\left||p_{j}|\right|}$, in order to put embedding vectors on the same footing for all functions. This is motivated by the expectation that isotropically distributed random gene embeddings (i.e. ‘noise’) should lead to the same distribution of $s_{ij}={\tilde{p}}_{j}\cdot x_{i}$ for all functions.

### 2.4 Gene–function prediction and prioritization

Signed causal gene–function relationships are predicted if the absolute value of the gene–function score defined by the scalar product $s_{ij}={\tilde{p}}_{j}\cdot x_{i}$ is greater than a certain threshold. For a given function, we can think of function embedding vectors ${\tilde{p}}_{j}$, based on the construction above, to be tilted toward ‘consensus’ sets of function-associated genes that have similar (or anti-similar) gene embedding vectors. This means that predicted genes that are also similar to one of these sets, as well as all genes within these sets (that are already known to be associated with the function), will receive high absolute scores. In this sense, scoring will prioritize ‘key’ genes that are concordant with the consensus sets. Likewise, genes whose embedding vectors are more scattered and not similar to one of the consensus sets, will not receive high scores, and thus not be prioritized. The choice of the embedding dimension *K* determines whether the gene–function prediction model tends to under- or overfit. If *K* is too small, not enough information will be encoded in the embedding vectors; if *K* is too large, the similarity between genes will not be sufficiently represented. For example, in the spectral model E1, in the limit *K *=* N* all gene embedding vectors are orthogonal.

Gene–function scores were also transformed to z-scores (see Supplementary data, Section 4). Since z-scores measure statistical significance, this is useful to define meaningful cutoffs for top-scoring genes.

### 2.5 Cosine similarity for embedded functions

The similarity of functions is determined by using cosine similarity of the associated embedding vectors, which in our case is simply given by their scalar product since function embedding vectors are normalized. This scalar product can assume negative values corresponding to ‘anti’-similarity, i.e. the activation of one function being similar to the inhibition of another. Statistical significance of function similarity can be assessed by considering the standard deviation *σ_c_* of the cosine similarity distribution (centered around 0) for two random unit vectors. Since one of these vectors can be held fixed, this is the same as the standard deviation of a single vector component *x_i_* of a random unit vector. From the condition $\sum_{i}x_{i}^{2}=1$ then follows that $1=\sum_{i}\langle x_{i}^{2}\rangle =K\sigma_{c}^{2}$ since all *K* vector components are equivalent. An appropriate significance threshold (at $2\sigma_{c}$) for the cosine similarity score is therefore $2K^{-1/2}$ which is about 0.09 for a typical embedding dimension of *K *=* *500.

### 2.6 Implementation

Algorithms were implemented in Python using the standard scientific computing stack (numpy, scipy, pandas, scikit-learn). Most code was run on a standard laptop in minutes to hours time frame. The implementation of the neural network-based embedding strategy E2 uses the pytorch framework, and we ran experiments on a machine with a T4 GPU (about 1 hour per run). For node2vec (E3) we utilized the python implementation provided by Grover and Leskovec (2016) based on the gensim library with default parameter settings (random walks with 30 nodes, 100 walks per node, hyperparameters p=q=1).

## 3 Results

### 3.1 Cross-validation of gene–function prediction

We used the following cross-validation approach to test the accuracy of gene–function prediction. We randomly set gene–function relationships *Y_ij_* to zero, trained the linear regression model and then determined how well those removed gene–function relationships could be predicted. To avoid artificial dependencies between functions, we included only ‘leaves’ of the function hierarchy in the subset of functions on which the model was tested and required that functions were supported by at least 10 genes. A balanced test set was created by randomly picking *n* entries of the matrix $Y=\{Y_{ij}\}$ that had the value 1, *n* entries that had the value −1, and 2*n* entries that were zero. We repeated the procedure *k* times to create *k* independent test sets. For each test set, the selected elements of *Y* were set to zero, and a model was trained using this new matrix *Y*. From the resulting gene–function scores, we then computed receiver-operating characteristic (ROC), and precision-recall curves (PRCs). Strictly speaking, zero-entries of *Y*, i.e. the lack of a gene–function relationship in the curated content are not true negative examples in a training or test set, since they do not mean that there was experimental evidence of no functional effect. However, we can assume that the vast majority of zero-entries in *Y* are true negative examples, and the few ‘false’ negative examples do not significantly affect test results.

Two prediction tasks were considered. For the first task, we predicted the presence of a gene–function relationship using an absolute gene–function score threshold |s| for the complete test set with 4n examples. For the second task, we used the signed score itself to predict the sign of the effect, i.e. whether it is activating or inhibiting, and the test set was limited to the 2*n* non-zero examples. There are two subcases corresponding to the prediction of either activation (versus inhibition) or inhibition (versus activation) among edges with unknown sign, which means there are two distinct PRCs. The ROC is symmetric w.r.t. these two subcases, i.e. the second subcase can be obtained from the first by transforming true (TPR) and false-positive rates (FPR) according to TPR →1− TPR, and FPR →1− FPR, or simply by ‘flipping’ the ROC curve.

Two metrics are used to assess the capability of our signed gene–function prediction model: The AUC, which measures overall how ranking by score discriminates between true positives and negatives, and the precision in the limit of low recall (here set to 5%) which measures how precise the predictions for the highest-scoring genes are. We use the latter metric because we are particularly interested in the identification of the most relevant, key genes causally affecting a given function or disease. In all cross-validation experiments, we set *n *=* *1000 and *k *=* *50.

**Fig. 2.**
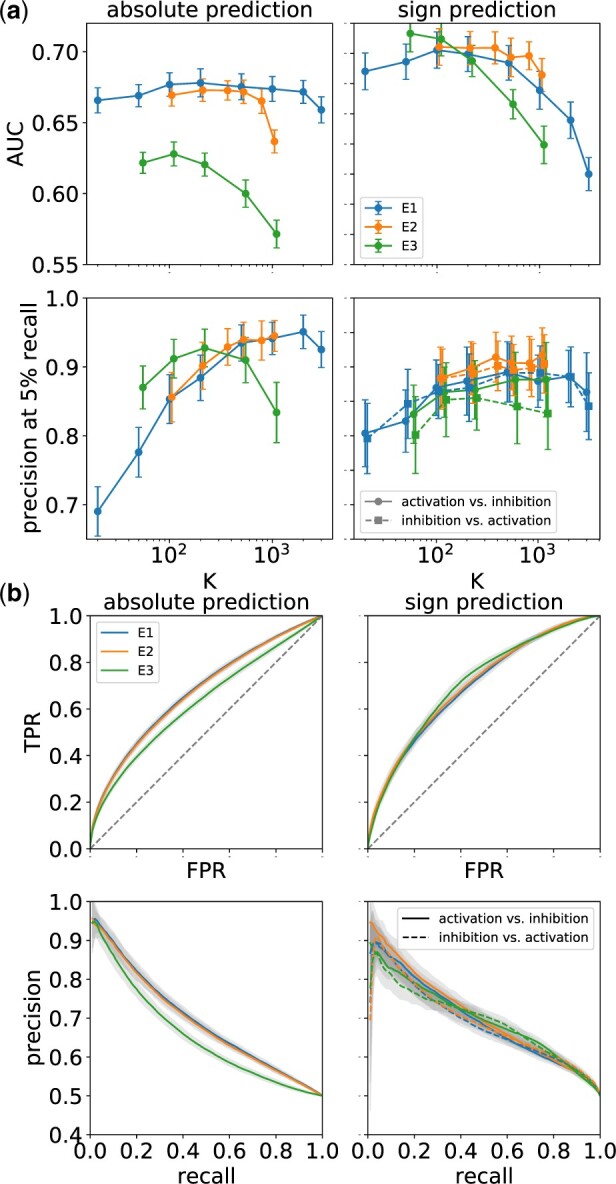
Cross-validation: (**a**) Average AUC and precision at 5% recall for absolute and sign prediction as a function of the embedding dimension *K* for models E1, E2 and E3. (**b**) ROC and PRCs for the cases *K *=* *500 (E1), *K *=* *350 (E2) and *K *=* *100 (E3). Error bars and shaded areas reflect standard deviations across 50 independent cross-validation runs

Figure 2a shows average AUC and precision at 5% recall for absolute and sign prediction as a function of the embedding dimension *K* for all models E1, E2 and E3. The neural network model E2 uses a single intermediate layer with $N_{2}=1000$ nodes, and the z-score cutoff for the graph-based model E3 was set to *z *=* *1.5. Error bars shown correspond to the measured standard deviation across the *k* replicated runs. We observed that increase of the number of nodes in the intermediate layer, or inserting an additional layer (E2) did not result in significant change, and larger cutoff values *z* lead to a decrease of AUC and precision (E3). From Figure 2a, one can obtain ‘optimal’ embedding dimensions for which AUC and precision are both large. Embedding dimensions greater than this optimal dimension will lead to over-fitting, while smaller embedding dimensions result in under-fitting of the model. This can be seen for all three cases, E1, E2 and E3, with slightly different behavior of AUC and precision curves. For the spectral case E1 (absolute prediction), the AUC curve shows a very broad peak with maximum AUC ≈0.68, while precision (at 5% recall) has a plateau around 95% for dimensions larger than 500, and drops sharply toward lower embedding dimensions. The behaviors of cases E1 and E2 are very close to each other (for absolute and sign prediction) with the AUC (for absolute prediction) dropping slightly more strongly toward high dimensions for the latter. For E3, performance is also similar except that the AUC is lower for absolute prediction, and the maximum (at AUC = 0.629) appears shifted to lower embedding dimensions likely because the model included many fewer genes, but it could also indicate a better representation compression. Figure 2b shows ROC and PRCs for the cases *K *=* *500 (E1), *K *=* *350 (E2) and *K *=* *100 (E3). All three models reach an average precision of nearly 95% for absolute prediction and about 90% for sign prediction, while the AUC for sign prediction is about 0.70. For the spectral approach, E1 we also evaluated models that require each included gene to have a minimum number of downstream-regulated genes in the bipartite graph *G* (see Supplementary data, Section 2.1).

Overall, we find that both the spectral model E1 and the neural network-based model E2 perform equally well in our cross-validation experiments, and both perform significantly better than the graph-based model E3 on the AUC for absolute prediction. Since embeddings for E2 are generally much more expensive to compute we therefore chose to concentrate on the spectral model E1 for some of the following applications. For the application to drug–disease prediction (see Section 3.4 below), we also performed a comparison between all three models.

As noted in Supplementary data, Section 1, there are about twice as many positive signs as negative signs in the bipartite graphs derived from the QKB. An interesting question is whether this imbalance has any effect on our results. This is discussed in Supplementary data, Section 2.2.

### 3.2 Function embeddings: discovery of latent biological relationships

The similarity of embedding vectors encoding functions and diseases is expected to reflect underlying biological relationships. In order to test this, we examined how functional contexts are represented in embedding space, constructed a global t-distributed stochastic neighbor embedding (tSNE) map of diseases and visualized relationships between diseases and associated biological functions (for the latter, see Supplementary data, Section 3).

**Fig. 3.**
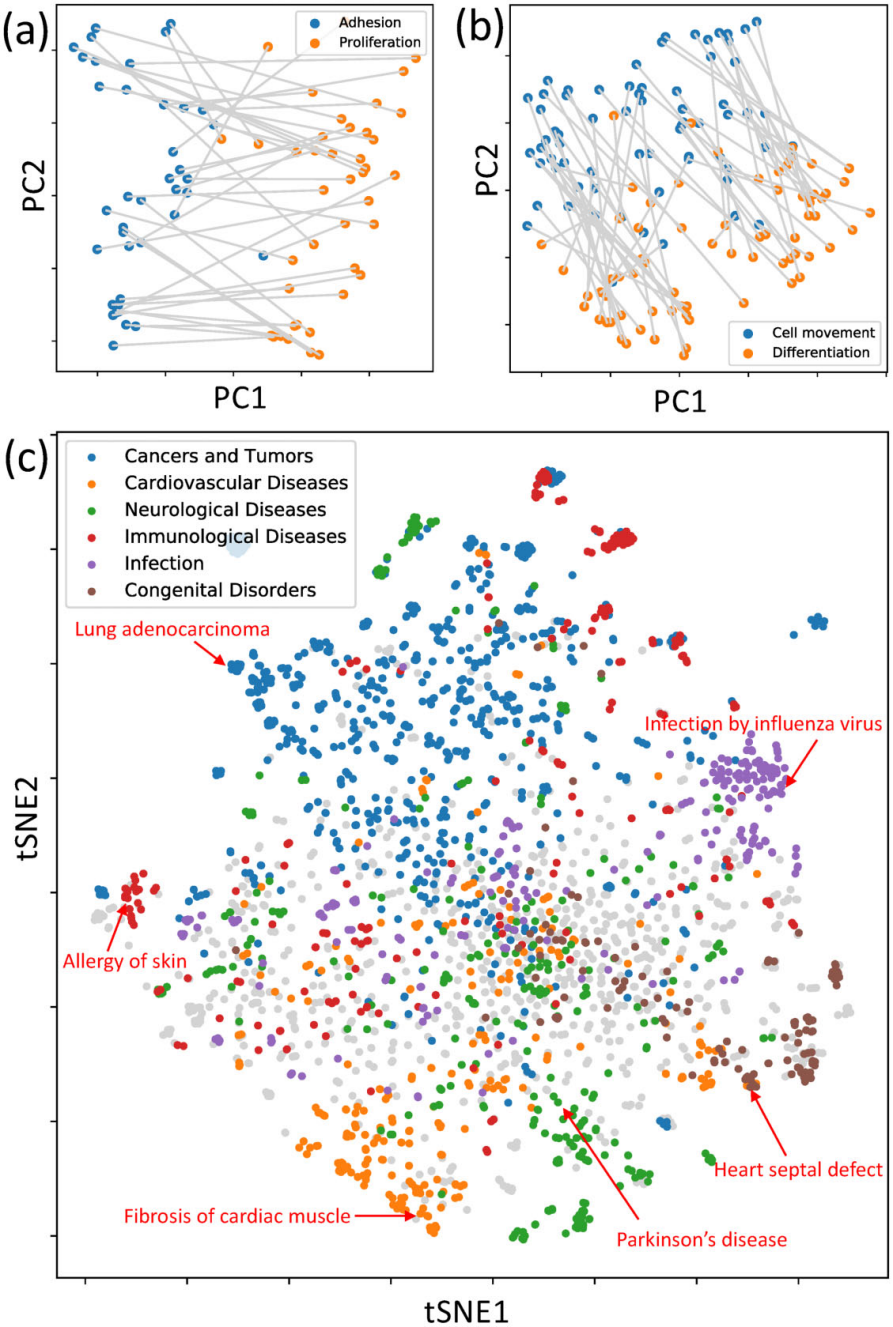
Discovery of latent biological relationships from function embeddings. (**a**, **b**) Two-dimensional projection of embedding vectors of functions of the form ‘X of Y’ where X is one of the biological processes *Adhesion*, *Proliferation*, *Cell movement* and *Differentiation*; and Y is one of the cell type contexts given in Supplementary Table S1 (e.g. *T lymphocytes*). (**c**) Global tSNE visualization of disease embedding vectors. Diseases from different disease categories (cardiovascular, neurological, immunological, infective, congenital or cancer) tend to cluster together. Note that cancer and the other disease categories are not exclusive, for instance, some cancers were also classified as immunological or neurological, and the non-cancer classification took precedence

One result of the word2vec algorithm (Mikolov *et al.*, 2013) is the association of semantic relationships with simple linear vector operations. For instance, in the most famous example, the vector representation of the word ‘king’ is related to the word ‘queen’ by the (approximate) identity ‘king’ = ‘queen’ – ‘female’ + ‘male’. In order to find similar relationships in our function embedding space, we consider functions that describe biological processes in a particular context. As an example, we examine functions of the form ‘X of Y’, where the biological process X is from the set *Adhesion*, *Proliferation*, *Cell movement*, *Differentiation*, and Y is a cell type (e.g. *T lymphocytes*, complete list given in Supplementary Table S1). Linear relationships between embeddings can be visualized by performing principal component analysis (PCA), and projecting embedding vectors on the two main principal components which are shown in Figure 3a and b for the process pairs *Adhesion versusProliferation*, and *Cell movement versusDifferentiation*. Pairs of functions with different processes, but the same cell type context are connected by straight line segments. If a linear vector relationship like in the ‘king’-‘queen’ example above holds, then these line segments are expected to be parallel. From Figure 3a and b, it is seen that this is approximately the case for most of the function pairs. In order to make a quantitative assessment of this observation, we computed the standard deviation of the distribution of angles that line segments form with the horizontal axis, and compared it to the standard deviation of angles of line segments with randomly shuffled endpoints. The resulting estimated *P*-values obtained by random sampling are $p=1\times 10^{-5}$ for the *Adhesion–Proliferation* pair, and $p=4\times 10^{-7}$ for the *Cell movement–Differentiation* pair, clearly showing the statistical significance of this result.

A global tSNE visualization of embedding vectors for diseases (after first reducing dimensionality to 20 using PCA) is shown in Figure 3c. It is seen that, except for the center of the tSNE map, diseases from the same disease category (cardiovascular, neurological, immunological, infective, congenital and cancer) tend to cluster together, indicating that function embedding vectors capture biological similarity and dissimilarity between diseases.

### 3.3 Application: inferred disease networks

To explore how the top-scoring genes for a given disease relate to its associated functions, we selected three examples, psoriasis, pulmonary hypertension and Alzheimer’s disease, which represent a wide spectrum of ‘systemic’ diseases with distinct underlying mechanisms and manifestations. For each of these diseases, we determined top-scoring genes and functions and their signs (see Supplementary Tables S2–S7). In order to give priority to the most ‘specific’ functions (rather than more general terms), we did not include functions that are parents in the process hierarchy of other functions in the list. Redundancy was further decreased by bundling functions from the same context (e.g. cell type), and considering only the highest scoring function from each bundle. For each disease, we constructed a bipartite graph connecting the 15 top-scoring genes and 20 top-scoring functions through edges if the absolute value of the corresponding gene–function score is greater than a certain threshold (here: |z-score|>3), and its sign is consistent with the signs of the adjacent gene and function.

**Fig. 4.**
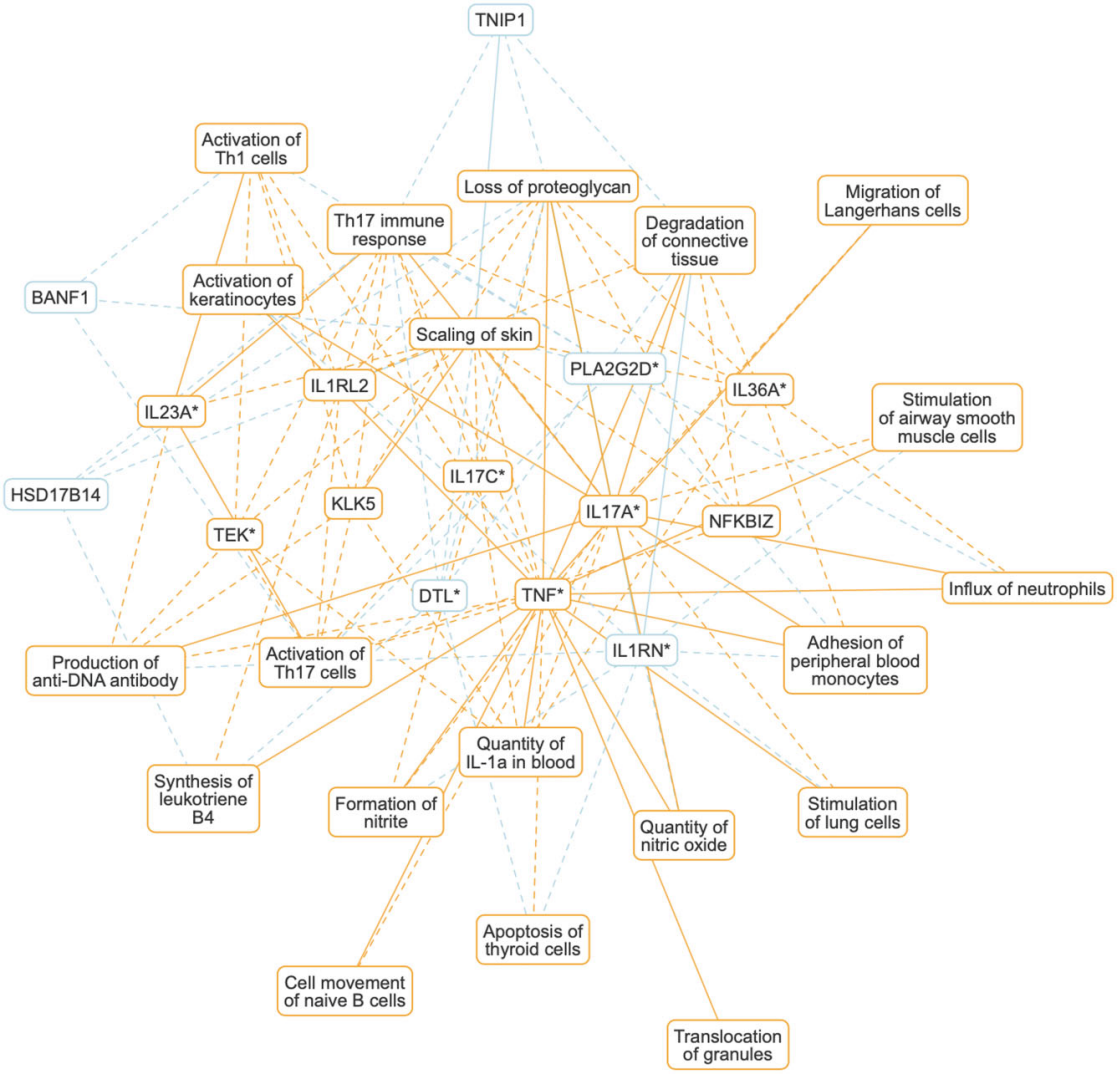
Psoriasis network. Bipartite graph connecting the 15 top-scoring genes and 20 top-scoring functions through edges with high absolute gene–function scores (|z-score|>3). The network shows disease-underlying biological functions and known disease genes, as well as genes that are predicted to be implicated in psoriasis based on QKB content. Each node (gene or function) carries a color-coded sign (positive: orange, negative: blue) depending on whether that gene or function is positively- or anti-correlated with psoriasis. The edge style indicates whether gene–function relationships are supported by content of the QKB (solid), or purely inferred (dashed). Genes marked with an asterisk (*) have known associations with psoriasis in the QKB

Figure 4 and Supplementary Figures S4 and S5 show networks constructed this way for all three diseases above. In the following, we discuss the psoriasis network. Similar discussions for the other two diseases are given in the Supplementary data (Section 4).

Psoriasis is a chronic inflammatory skin disease with a strong genetic component (Greb *et al.*, 2016). The disease has multiple forms and also may affect organs other than the skin. The network shown in Figure 4 highlights the main immune axis represented by the IL17-IL23 T helper components (*Activation of Th1 cells*, *Activation of Th17 cells*). IL17 and IL23, as well as TNF, are known to be involved in the pathogenesis of psoriasis. One of the hallmarks of psoriasis is keratinocyte proliferation and immune cell infiltration. This and the disease phenotype (*Scaling of skin*, *Degradation of connective tissue*) are well represented among the functions shown in the network (*Activation of keratinocytes*, *Adhesion of peripheral blood monocytes*, *Cell movement of naive B cells*, *Influx of neutrophils*, *Migration of Langerhans cells*). A number of genes shown are purely predicted from QKB content (BANF1, HSD17B14, IL1RL2, KLK5, NFKBIZ and TNIP1). An independent literature search uncovered known or suspected involvement of these genes in the disease: BANF1 has been suggested to be associated with increased proliferation of keratinocytes in psoriatic lesions (Takama *et al.*, 2013). Kallikreins (like KLK5) were found in the serum of patients with psoriasis which suggests that they might be involved in the pathogenesis (Komatsu *et al.*, 2007). The expression of NFKBIZ (a nuclear inhibitor of NF-*κ*B) in keratinocytes has been found to trigger not only skin lesions but also systemic inflammation in mouse psoriasis models (Lorscheid *et al.*, 2019). Loss of TNIP1 in keratinocytes leads to deregulation of IL-17-induced gene expression and increased chemokine production *in vitro* and psoriasis-like inflammation *in vivo* (Ippagunta *et al.*, 2016).

This demonstrates that these networks indeed capture known underlying disease mechanisms and also have the potential to generate novel insights.

### 3.4 Application: drug–disease prediction

**Fig. 5.**
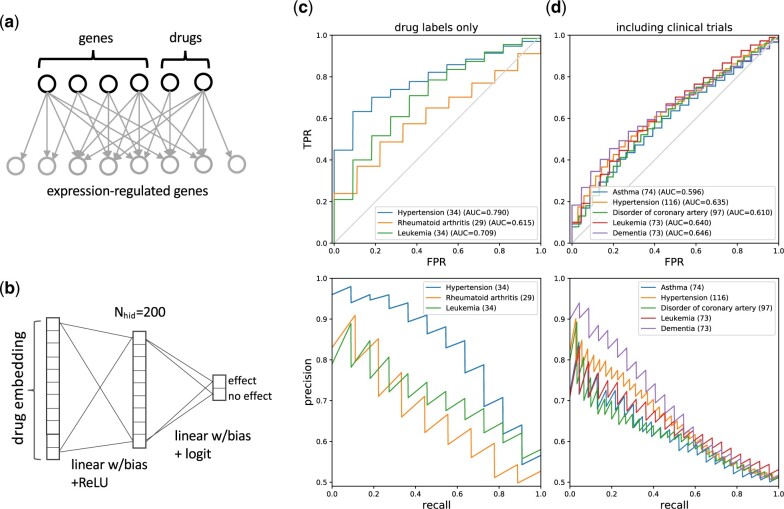
Drug–disease prediction. (**a**) Expression bipartite graph including genes and drugs. (**b**) MLP trained on drug labels and/or clinical trial information to predict drug–disease associations using feature representations based on drug embedding vectors. The MLP used here has one hidden layer with 200 nodes. (**c** + **d**) Average ROC and PRCs for drug–disease prediction experiments for several diseases. (c) MLP trained on drug labels (approved drugs). (d) MLP trained on drug labels and clinical trial drugs. Shown in parentheses are the numbers of existing drugs targeting the respective disease

In the following, we demonstrate that the embeddings computed with our approach can also be used for independent prediction tasks. As an example, we consider the prediction of drug effects on diseases. Since the QKB also contains literature-derived information about the effect of drugs on gene expression, it is straightforward to extend the gene embedding model also to drug molecules by simply adding them to the expression bipartite graph G (Fig. 5a). In total, we included 1111 drugs for which embedding vectors were computed.

Using known drug indications for a given disease, we train a simple multilayer perceptron (MLP) by employing drug embeddings as feature vectors (Fig. 5b), and then assess how well this model performs in predicting new drug–disease associations. Known drug–disease relationships used for this purpose were curated (as part of the QKB) from drug labels (approved indications) and phase 3 and 4 clinical trials, which is described in more detail in Supplementary data, Section 5.1.

Here, we only focus on diseases that are associated with a sufficient number of approved drugs or drugs in clinical trials. For training the MLP (for a given disease), those drugs are used as positive examples, while a set of negative examples is randomly drawn from all the other drugs. Both sets of positive and negative examples are equal in size to create balanced training and test sets, utilizing a 70/30 random split for cross-validation. Training and testing are then repeated 100 times to compute average ROC and PRCs.

Results are shown in Figure 5c and d for the spectral model E1 (*K *=* *500) for several diseases using either only approved drugs, or also including drugs in clinical trials. It is seen that the performance in the first case is generally better than in the second (e.g. AUC = 0.790 versus AUC = 0.635 for Hypertension) which may be caused by approved drugs being more similar to each other than the larger set of drugs in clinical trials, thus leading to a more coherent predictive model. Overall, it is seen that drug embedding vectors, obtained from literature-curated causal gene expression relationships indeed capture information about drug effects on diseases. For comparison, we have also performed the drug–disease prediction experiments for the other models E2 and E3 (see Supplementary data, Section 5.2).

It shall be noted that one limitation of these results is the sparsity of the training data, i.e. only a few diseases are targeted by a sufficient number of drugs to perform a meaningful split into training and test sets. Also, no additional effort was made in the selection of included drugs other than their approval status or inclusion in a clinical trial. We did not distinguish between drugs that have very general indications to manage symptoms and others that have not.

### 3.5 Comparison to gene embeddings based on other information

We compared our gene embeddings to those obtained with gene2vec (Du *et al.*, 2019; based on coexpression) and Mashup (Cho *et al.*, 2016; based on protein–protein interactions). For the gene–function prediction task (Section 3.1), we find that our approach outperforms gene2vec, while performing at the same level as Mashup. We also find that top-scoring gene sets computed with our approach are mostly disjoint from those computed with Mashup. For a discussion, see Supplementary data, Section 6.

## 4 Discussion

We have used signed cause–effect relationships curated from the biomedical literature to construct high-dimensional embeddings of genes, biological functions and diseases. Gene embeddings are based on literature-derived downstream expression signatures in contrast to embeddings obtained with existing approaches that leverage either coexpression, or protein binding networks. Function embeddings are constructed using gene embedding vectors with a linear model trained on signed gene–function relationships.

Three separate methods were applied to construct gene embeddings, a ‘spectral’ approach based on a low-rank matrix approximation, a neural network-based approach to capture non-linear effects and a graph-based method utilizing the node2vec algorithm. All three methods performed similarly, reaching on average close to 95% precision for top-scoring genes (90% precision for distinguishing between activating and inhibiting effects) in cross-validation experiments for the gene–function prediction task.

By analyzing various examples, we showed that function embedding vectors capture hidden biological relationships as well as semantic context similar to word embeddings. As an application, we determined top-scoring genes and related functions for three diseases, Alzheimer’s disease, pulmonary hypertension and psoriasis, to build disease-specific networks. These networks show key genes known to be involved in disease progression, and they capture underlying cellular and physiological processes. We were able to predict a number of disease genes that were not present in the training data (i.e. connected to the disease in the QKB) but could be validated through an independent literature search. It shall be noted that a current constraint of our method is that only a fraction of genes (≈30%) can be covered, limited by content curation and available literature coverage.

In order to demonstrate the applicability of our approach to other prediction tasks, we extended gene embeddings also to drug molecules and used a simple MLP, trained on known drug–disease associations from drug labels and clinical trials, to predict new drug–disease associations. We find that drug embedding vectors, obtained from literature-curated causal gene expression relationships indeed capture information about drug effects on diseases.

Our work illustrates that prior knowledge from the biomedical literature can be used collectively to generate new insights, going beyond the findings reported in individual research articles. Applications of knowledge-driven embedding models are manifold. As already implied by the disease networks discussed here, the approach can be used to create new hypotheses for biological mechanisms, identify new potential gene targets for drug repurposing or predict possible new disease indications in a given therapeutic context.

## Supplementary Material

vbac022_Supplementary_Data
