# Supplementary material for: Mining hidden knowledge: embedding models of cause–effect relationships curated from the biomedical literature
Source: Bioinform Adv. 2022 Apr 7;2(1):vbac022. doi: 10.1093/bioadv/vbac022 (PMC9710590; doi:10.1093/bioadv/vbac022)
Supplement: vbac022_Supplementary_Data [file vbac022_supplementary_data.zip › supplementary_data.pdf]

Supplementary Data

Mining hidden knowledge: Embedding models of cause-effect relationships curated from the biomedical literature

A. Krämer, J. Green, J.-N. Billaud, N. A. Pasare, M. Jones, S. Tugendreich

1.1. QKB content

1.1. Summary statistics

The knowledge graph obtained from the QKB and used for this paper contains in total 6,757 genes that appear in both literature-derived gene expression and gene-function relationships. The total number of included expression edges is 147,792 with 286,022 underlying literature findings, and 14,176 regulated genes. There are 217,239 gene-function edges with 395,224 underlying findings that regulate 29,553 functions of which 7,388 are diseases. As part of an ontology, functions are organized in a hierarchy where, except for very general terms, parents inherit causal gene associations (and edge signs) from their descendants. This inheritance mechanism increases the total number of gene-function edges to 748,626. The sign distribution on edges is slightly unbalanced, with roughly two thirds of edge signs being positive, and one third of edges being negative (for both, gene expression, and gene-function edges).

In total, 92% of findings used in this work are manually curated from the biomedical literature using the full text of articles including figures and tables. The remaining 8% originate from various sources and databases, including GO, clinicaltrials.gov, and DrugBank. Genes are mapped to ortholog clusters (using HomoloGene) and cover the species human, mouse, and rat.

1.2. Examples

The following table shows examples for gene expression (E) and gene-function findings (P) representing experimentally observed causal effects from the literature. The textual description frequently also contains experimental context and other details. These details are used to determine the proper edge sign, for instance a finding “Inhibition of A increases expression of B” translates to the edge “A -| B”. Here, and in the table below, “-|” stands for inhibition, and “->” for “activation”. Note, that expression findings are not based on high-throughput expression data but represent individual reported observations of causal effects on the expression of single genes. Experimental context (e.g. tissue or cell line) is ignored when mapping to graph edges.

	signed edge	edge type	description	PubMed ID
0	FOXA1 -| MALT1	E	Interference of human FOXA1 mRNA by siRNA increases expression of human MALT1 mRNA in Sk Br 3 cells.	22391567
1	HIF1A -> NEK8	E	Interference of human HIF1A mRNA by siRNA decreases expression of human NEK8 protein in CaKi cells that is increased by hypoxia.	25451921
2	ADORA2A -> CKB	E	In mouse, homozygous mutant mouse A2aR [Adora2a] gene (knockout) decreases expression of mouse Ckb mRNA in mouse striatum.	16046619
3	PAX3 -> EYA2	E	PAX3 protein increases expression of mouse Eya2 mRNA.	11262400
4	NOTCH1 -| MMP3	E	A protein fragment (1758-2556) containing a intracellular domain from human NOTCH1 protein decreases expression of human MMP3 mRNA in senescent Imr 90 cells.	27525720
5	EP300 -> CPT2	E	In heart from 4 week-old mouse, dominant negative transgenic mutant P300 [EP300] protein (deletion with its C/H3 domain deleted) decreases expression of mouse CPTII [Cpt2] mRNA in heart ventricle from 4 week-old mouse.	19729597
6	HRAS -> PSMB9	E	In NIH/3T3 cells, mouse Ifn gamma [Ifng] protein increases expression of mouse Lmp2 [Psmb9] mRNA that is increased by transgenic RAS [HRAS] protein.	9485192
7	CTNNB1 -> BGLAP	E	Interference of mouse beta-catenin [Ctnnb1] mRNA by siRNA decreases expression of mouse Ocn [Bglap] mRNA in MC3T3-E1 cells that is increased by melatonin.	31216173
8	RANBP9 -> FOS	E	Human RANBPM [RANBP9] protein increases activation of a luciferase reporter gene with a DNA endogenous promoter from the C-FOS [FOS] gene that has a serum response element.	12147692
9	NGF -| EGLN3	E	Withdrawal of Ngf protein increases expression of rat Egln3 mRNA in sympathetic neurons from rat superior cervical ganglion.	20702711
10	TP53 -> CSF1R	E	Mouse p53 [Trp53] protein is necessary for expression of mouse M-csfr [Csf1r] mRNA that involves gamma radiation.	9764819
11	HIF1A -> Production of lactic acid	P	Mutation of mouse Hif1a gene to homozygous mutant mouse Hif1a gene (knockout) in mouse decreases production of lactate [lactic acid] in cultured mouse BMDM [bone marrow-derived macrophages] infected by Mycobacterium tuberculosis strain Erdman that involves mouse Ifng protein.	27430718
12	HTT -> Stereotypy	P	A transgenic protein fragment containing a exon 1 and a CAG repeat (115-156 repeats) from HD [HTT] protein in mouse increases stereotypy by mouse.	10515664
13	PRSS12 -> Sarcopenia of extensor digitorum longus muscle	P	In motoneurons from spinal cord of young adult mouse, transgenic PRSS12 protein increases sarcopenia of extensor digitorum longus muscle in young adult mouse.	21885656
14	RAB27A -> Cytotoxicity of T lymphocytes	P	In T lymphocytes, mutant RAB27A gene (unspecified knockout) decreases cytotoxicity of T lymphocytes.	14745138
15	THPO -> Activation of blood platelets	P	Thrombopoietin [THPO] increases activation of human platelets [blood platelets].	9459347
16	PCDHGC5 -| Apoptosis of neurons	P	Mouse Pcdhgc5 decreases apoptosis of neurons.	32633719
17	RAG1 -> Degeneration of myelin sheath	P	In mouse, homozygous mutant mouse Rag1 gene (knockout) decreases degeneration of myelin sheath that is increased by heterozygous mutant mouse Mpz gene (knockout).	10632602
18	STAM -| Gliosis	P	In 129S4/SvJae * C57BL/6 mouse, mutant mouse Stam gene (allele Stamtm1Sug/Stamtm1Sug) (knockout [homozygous]) increases gliosis in mouse.	11340172
19	SCTR -> Concentration of cyclic AMP	P	In the intracellular space from mammal bile duct cholangiocytes, dietary administration of alpha-naphthylisothiocyanate [1-naphthylisothiocyanate] (chronically) and SCTR protein increase quantity of cAMP [cyclic AMP].	11318975
20	EP300 -> Initiation of transcription of RNA	P	In a cell-free system, human p300 [EP300] protein and human SRC1 [NCOA1] protein increase the initiation of transcription of gene.	11606780
21	EP300 -> Apoptosis of tumor cells	P	In tumor cells, E2F1 protein and P300 [EP300] protein increase apoptosis of tumor cells that involves homozygous mutant P53 [TP53] gene (knockout).	9652736

1.3. Confidence

We have high confidence in the validity of curated findings from the peer-reviewed literature, representing individually published experimental observations. Therefore, we don't use a confidence measure on the single-finding level. All edges generally bundle a number of underlying literature findings from various experimental contexts, and edge signs reflect a consensus among all those contexts. While we occasionally find discrepancies between the signs of individual findings underlying an edge (because of different contexts or other possible errors), this is rare. An analysis shows that on the average only 5% of single-finding signs deviate from the consensus. If edges cannot be assigned an unambiguous sign, they are excluded. We do not explicitly down-weight relationships that are only supported by one or few observations compared to those that are supported by many. We expect that this may lead to some increased noise for those edges that have only little support. However, weighting would introduce bias against areas of biological research that are less studied than others which we want to avoid.

2. Cross-validation

2.1. Cross-validation for smaller networks

For the spectral model E1 we also built models that require each included gene to have a minimum number N_{min} of downstream regulated genes in the bipartite graph G. The number of embedded genes included in these models decreases strongly with increasing value of N_{min} which is shown in the inset of Figure S1. Because the spectral model is linear, it is expected that the optimal embedding dimension scales linearly with the number of genes (i.e., the size of the matrix S). This is confirmed by overlaying scaled AUC-vs-dimension and precision-vs-dimension functions in one plot showing an approximate collapse onto one curve (see Figure S2). Figure S1 shows AUC and precision (at 5% recall) plotted against the parameter N_{min} for these optimal embedding dimensions. It is seen that the AUC for absolute prediction decreases for increasing N_{min} because functions are represented by fewer genes and therefore embedding vectors carry less information. At the same time the AUC for sign prediction increases, presumably because only genes are included that are encoded based on a greater number of downstream expressed genes in G, thus reducing noise.

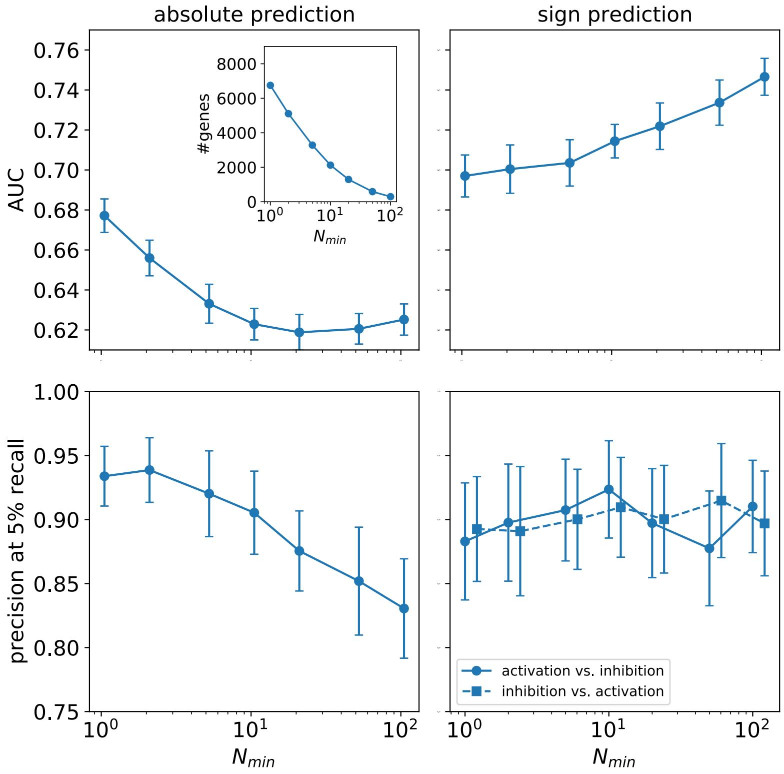

Figure S1. Cross validation: AUC and precision (at 5% recall) plotted against the parameter N_{min} for optimal embedding dimensions (spectral model). Error bars correspond to the measured standard

deviation across the 50 replicated runs. The inset shows the number of genes included in the model as a function of N_{min}.

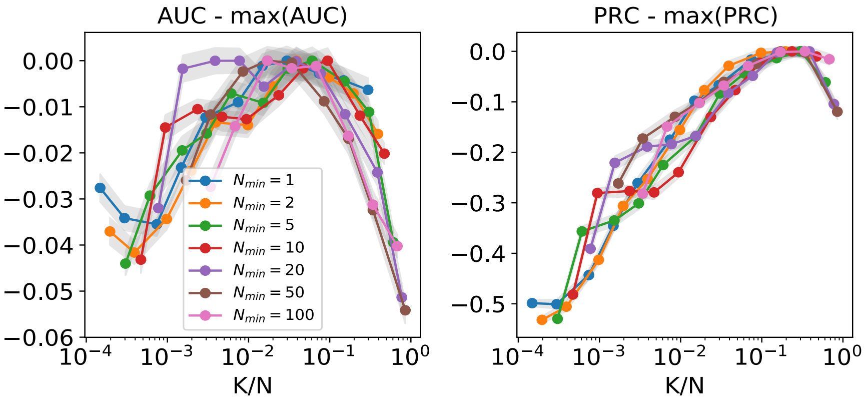

Figure S2. Scaling plots. Because the spectral model is linear, it is expected that the optimal embedding dimension scales linearly with the number of genes. This is confirmed by plotting AUC and precision at 5% recall as function of K/N (where K is the embedding dimension and N is the total number of genes) for models with different parameters N_{min} and also subtracting their maximal value. It is seen that the resulting curves for each model approximately collapse onto one curve.

2.2. Effect of the imbalance between positive and negative edge signs

As noted in Section 1 above, there are about twice as many positive signs as negative signs in the bipartite graphs derived from the QKB. An interesting question is whether there is any effect of the imbalance between positive and negative edge signs on our results. That this is not the case can be seen in the overall distribution of gene-function scores shown below. For all three models E1, E2, and E3, these distributions, while different, are all strongly peaked at zero and not visibly skewed. Note, that we use a logarithmic scale here (e.g. as a reference, for a Gaussian peak we would expect an upside-down parabola), and different magnitudes of scores between the three models only reflect irrelevant overall scale factors of the embedding vectors. At least for the model E1 the initial positive/negative imbalance of 2:1 is visible in the far tails of the distribution: For example, we find in total 140 instances of a positive score being greater than 0.26, and 65 instances of a negative score being smaller than -0.26.

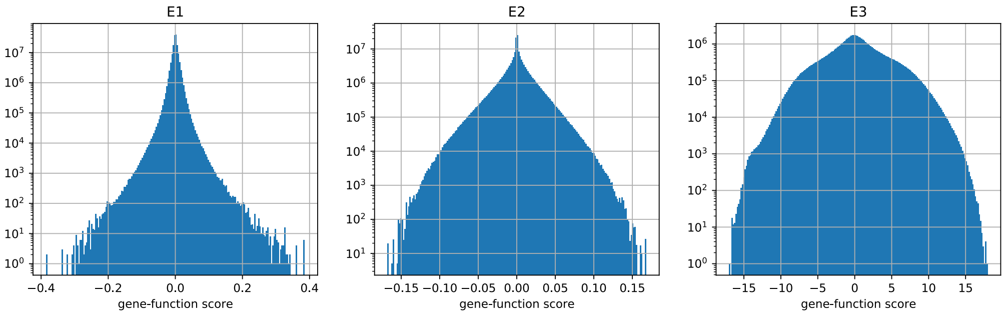

The precise centering of the distribution functions around zero can be explained by the sparsity of the gene-function bipartite graph, i.e., by the fact that there are about 1000 times more zero entries of the matrix *Y_ij* (see main text, Section 2.3) than there are positive and negative entries. This majority of zero entries dominates the symmetric linear regression problem used to compute the function embedding vectors, so that the imbalance between positive and negative signs does not matter.

3. Visualization of relationships between a disease and associated biological functions

The neighborhood in embedding space around a given disease can be mapped onto the two top PCA components, where PCA in this case is performed on all functions in the vicinity of the disease, as determined by a preselected cut off on the absolute value of the cosine similarity (here: 0.2). Before performing PCA, we multiply with -1 all function embedding vectors whose cosine similarity is less than zero; those functions *f* are therefore read as "inhibition of *f*" or "decrease of *f*". This allows for functions that are related but have an opposite sign to appear close to each other in the PCA projection. The result is shown in Figure S3 for the example of Alzheimer's disease (AD), where related functions whose sign was inverted are shown in blue, and all others in red. For better readability, Figure S3 shows a subset of all functions, similar to AD, where redundant other functions were removed. This is described in more detail in section 3.3 (main text). Interestingly, this procedure automatically detects many of the underlying disease manifestations of AD which are purely inferred from our embedding model of genes and functions. No explicit function-function or function-disease relationships from the literature were used in this approach. Note, that predicted functions potentially reflect underlying disease mechanisms, however, this cannot be distinguished from processes that share biological aspects of the disease but are not directly involved in it.

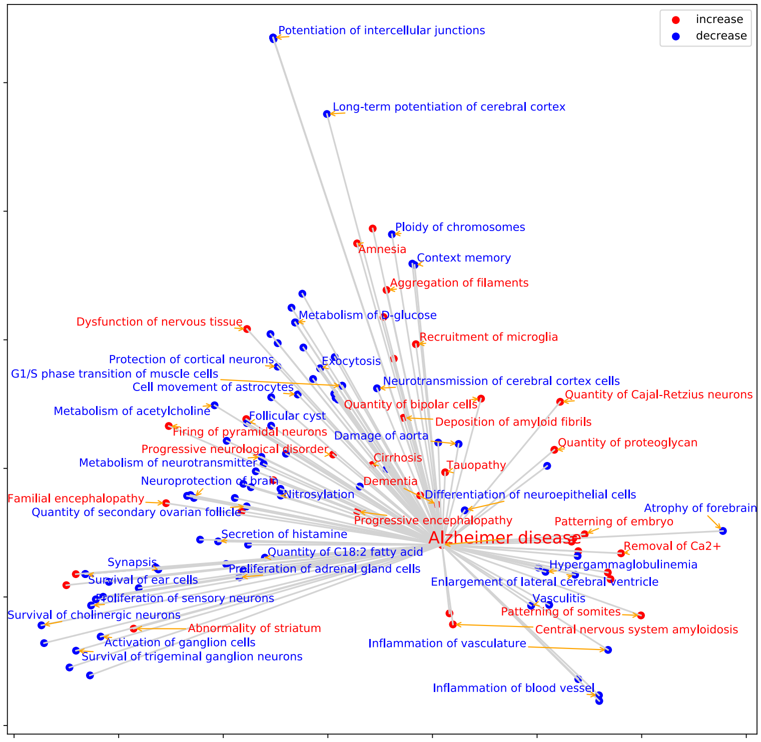

Figure S3. PCA projection of functions and diseases in the neighborhood of "Alzheimer's disease". Functions with embedding vectors that are anti-similar to Alzheimer's disease are shown in blue (with the embedding vector multiplied by -1), vectors that are similar to Alzheimer's Disease are shown in red.

4. Disease networks

For the following, gene-function scores s_{ij} were transformed to z-scores,

$z_{ij} = \frac{s_{ij} - mean_i(s_{ij})}{std_i(s_{ij})}$ that normalize the distribution of scores s_{ij} for each function j independently. Since z-scores measure statistical significance, this is useful to define meaningful cut offs for top-scoring genes (e.g., $|z|>2$). We verified that z-scores, and the original gene-function scores s are linearly related with approximately the same scale factor for all functions, so they can be used interchangeably.

4.1. Alzheimer’s disease

AD is a progressive neurodegenerative disease characterized by severe cognitive impairment, progressive extensive neuronal death, and eventually severe dementia [1, 2, 3]. Neuronal death is one of the main histological and biological markers of AD with hippocampus and striatum being CNS targets, which is reflected in several inhibited functions (i.e., with negative sign) shown in the network in Figure S4 (*Activation of spinal neuron*, *Cell viability of striatal neurons*, *Survival of trigeminal ganglion neurons*, *Chemotaxis*

of axons, *Synaptic transmission of hippocampal neurons*, *Metabolism of acetylcholine*). Other functions present in the network are *Amyloidosis*, *Aggregation of filaments*, and *Quantity of proteoglycan* (abnormal amyloid peptide aggregation is a histopathological hallmark of AD), decrease of *Spatial learning* (reflecting cognitive impairment), *Inflammation of vessel* (one of the hallmarks of AD is modification of the cerebral vasculature [4]), inhibition of *Metabolism of D-glucose* (evidence suggests that glucose hypometabolism may be a key player in dementia pathology [5]), and *Acidification of lysosome* (AD is associated with autophagy anomalies, and defective lysosomal acidification contributes to proteolytic failure [6]). The network contains a number of genes that have been implicated in AD and are represented in the QKB (APOE, APP, BDNF, HMGCR, INS, NGF, PSEN1, PSEN2), as well as others that are predicted (CUX2, FBXL7, HRG, LOX, PRR5, SLITRK5, Slfn1). Among the predicted genes, SLITRK5 has no known association with AD per se, but may participate in disease progression through intermediate connections. In fact, SLITRK5 indirectly modulates BDNF, and has a known role in other neurological disorders [7]. Another predicted gene, LOX (lipoxygenase) is known to promote neuroinflammation, and is regarded as a promising therapeutic target for AD [4].

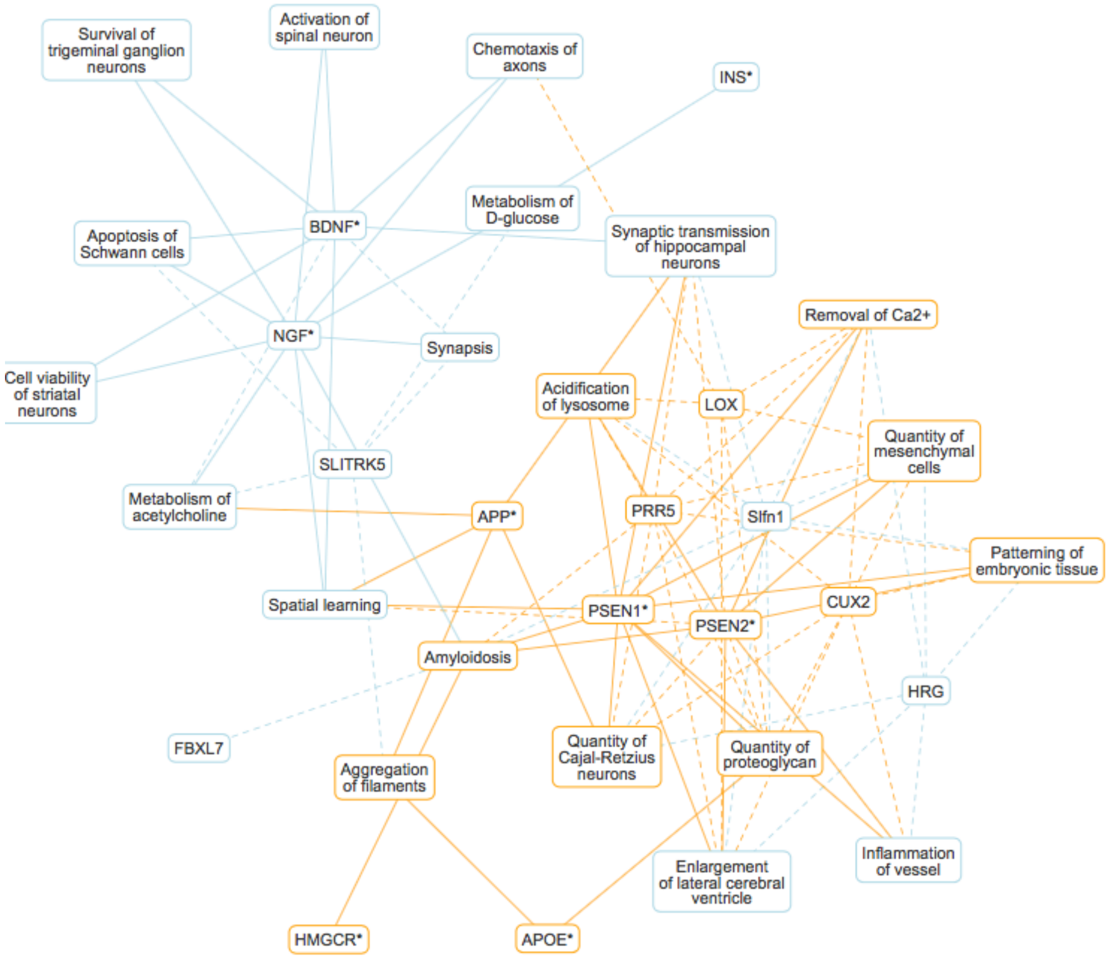

Figure S4. Alzheimer's disease network. Bipartite graph connecting the 15 top-scoring genes, and 20 top-scoring functions through edges with high absolute gene-function scores (|z-score| > 3). The network shows a number of disease-underlying biological functions and known disease genes, as well as genes that are predicted to be implicated in AD based on QKB content (see detailed discussion in main text). Each node carries a color-coded sign (positive: orange, negative: blue) depending on whether that gene or function is positively- or anti-correlated with Alzheimer's disease. The edge style indicates whether gene-function relationships are supported by content of the QKB (solid), or purely inferred (dashed). Genes marked with an asterisk (*) have known associations with Alzheimer's disease in the QKB.

4.2. Pulmonary hypertension

Pulmonary hypertension (PH), especially pulmonary arterial hypertension develops after the resting threshold pressure in pulmonary arteries is exceeded, typically resulting in right ventricular dysfunction and failure, and often leading to death [8]. PH induces vascular remodeling characterized by production of new endothelial cells, myofibroblasts, vascular smooth cells, extracellular matrix changes and fibrosis induction. The network shown in Figure S5 reflects this through appearance of the functions *Systolic pressure of right ventricle*, *Muscularization of artery*, and *Pulmonary fibrosis*. We also observe roles of immune cells (bone marrow-derived dendritic cells, T lymphocytes, mast cells, macrophages, and others) which are present in vascular lesions in patients with PH [9]. In particular, the recruitment of macrophages in perivascular regions of pulmonary arteries has been observed [10]. A number of genes present in the network have a known association with PH as represented in the QKB (ADA, ADORA2B, APOE, ARG1, BMPR2, IL18, IL1RN, PTGDR2, RPTOR). Among these is ADORA2B, which through its effect on the Pulmonary fibrosis or aplastic anemia appears to mediate the development of PH, and which is regarded as a potential therapeutic target [11, 12]. BMPR2 is also a major player in PH [13], as mutations in the gene have been identified as the main genetic cause [14, 15]. The network indicates the contribution of these two proteins towards increasing the systolic pressure of the right ventricle. The network also contains several predicted genes (ACVR2A, AKNA, AQP11, CCR3, IL15, P4HTM) that are not associated with PH in the QKB. An independent literature search found that aquaporins (AQP0-12) may be involved in PH under hypoxic conditions [16], and ACVR2A is a type 2 BMP receptor like BMPR2.

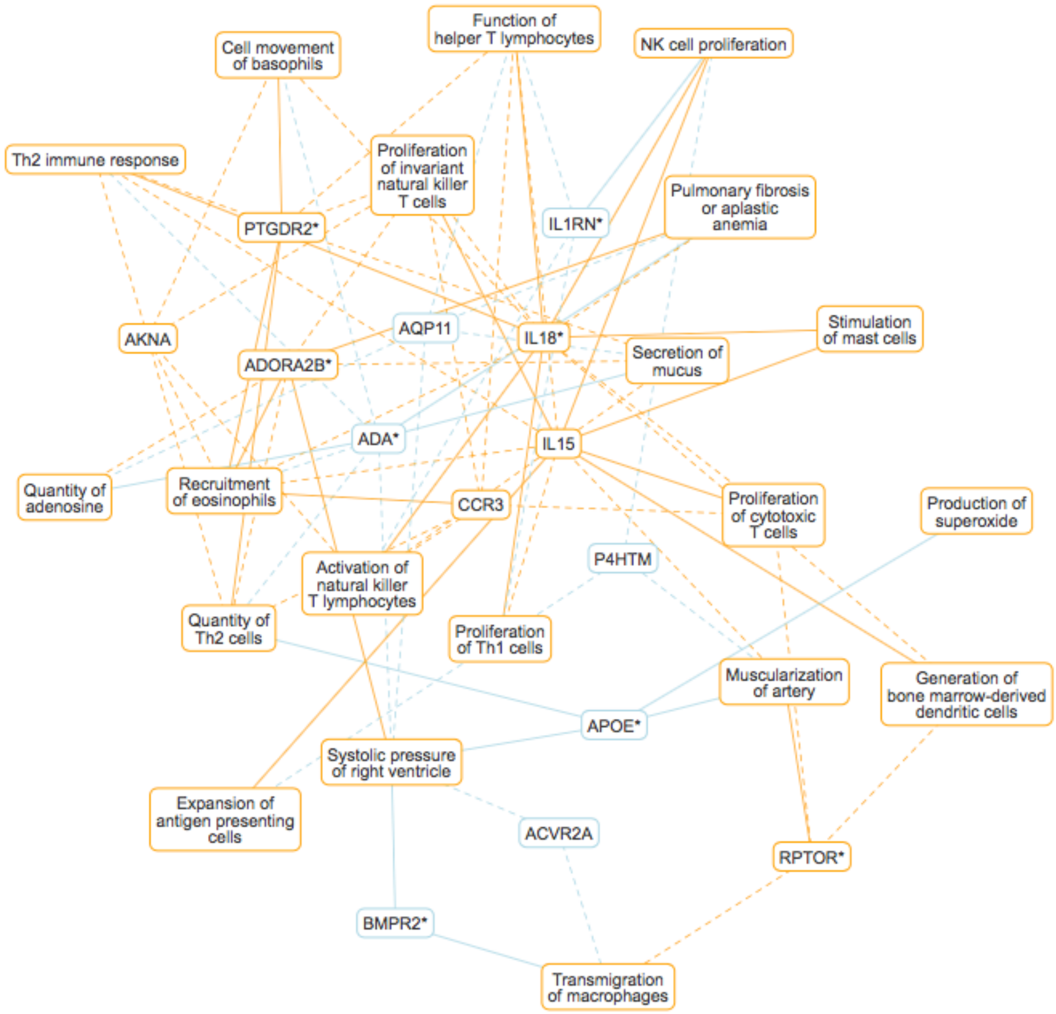

Figure S5. Pulmonary hypertension network. Bipartite graph connecting the 15 top-scoring genes, and 20 top-scoring functions through edges with high absolute gene-function scores (|z-score| > 3). The network shows a number of disease-underlying biological functions and known disease genes, as well as genes that are predicted to be implicated in PH based on QKB content (see detailed discussion in main text). Each node (gene or function) carries a color-coded sign (positive: orange, negative: blue) depending on whether that gene or function is positively- or anti-correlated with pulmonary hypertension. The edge style indicates whether gene-function relationships are supported by content of the QKB (solid), or purely inferred (dashed). Genes marked with an asterisk (*) have known associations with pulmonary hypertension in the QKB.

5. Drug-disease predictions

5.1. Drug-disease associations

As part of the QKB, drug-disease associations are curated from drug labels and clinical trials. For drug approvals we capture and update content from the Food and Drug Administration (FDA) and the European Medicines Agency (EMA) for all indications, and from the Pharmaceutical and Medical Devices Agency (PMDA) for oncology indications. For clinical trials content is obtained from ClinicalTrials.gov which is either imported automatically or manually curated. Manual curation is focused on trials that are recruiting and which have oncology indications. Drugs and diseases need to be able to be mapped to entities in the QKB. No further selection of drug-disease associations was done beyond this mapping requirement.

5.2. Comparison between models E1, E2, E3

Below we show a comparison of the computed AUC values for the three embedding models E1, E2, and E3 for diseases with the largest numbers of associated drugs (numbers given in parentheses). For the drug labels-only prediction all three models perform similarly on the average with some variation. When also drugs from clinical trials are included, interestingly we find a slightly better performance for the node2vec model E3. We suspect that the relatively high AUC values for Hypertension and Depressive disorder is caused by the high similarity of the involved drugs.

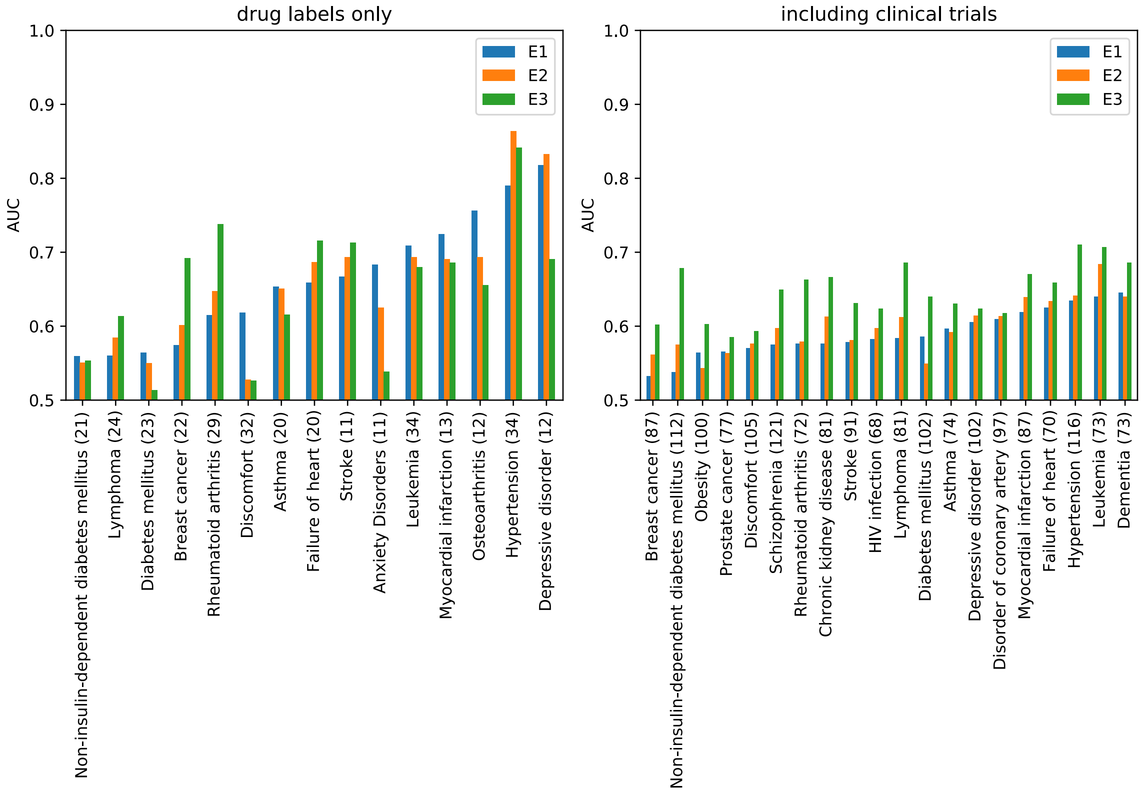

6. Comparison to gene embeddings based on other information

In order to compare our causal expression-based gene embeddings to other gene embedding approaches, we downloaded pre-trained gene embedding vectors generated with both, the gene2vec algorithm (https://github.com/jingcheng-du/Gene2vec), and Mashup (http://cb.csail.mit.edu/cb/mashup). Gene2vec (Du *et al.*, 2019) uses gene co-expression patterns from 984 GEO datasets to construct 200-dimesional vector representations of human genes by training a 3-layer neural network with gene pairs that are highly co-expressed. Mashup (Cho *et al.*, 2016) is based on a network diffusion approach that performs random walks with restart, and computes a lower-dimensional approximation of diffusion states. Here, we used the pre-computed, 800-dimensional embedding vectors for human genes based on protein-protein interactions from the STRING database [20]. We tested the gene2vec and Mashup embedding vectors on the same gene-function prediction tasks and cross-validated as described in Section 3. These tests were performed on the intersection of genes included in gene2vec (or Mashup) and our spectral model (gene2vec: 6,187, Mashup: 5,689), and for comparison we also reran tests for our model only including genes in the intersection.

For the absolute prediction task gene2vec reaches an AUC of 0.536 and precision at 5% recall of 0.647 compared to values of 0.677 and 0.934 in our model. The superior performance of our model shows that gene encodings based on causal expression responses likely contain more information about gene function than encodings based on co-expression. For Mashup we obtain an AUC of 0.663 and precision at 5% recall of 0.972, i.e. a performance similar to our model (AUC=0.672, precision at 5% recall=0.927). Performance for the sign prediction task was almost identical for both models. This indicates that causal gene expression and protein-protein interactions are on the average equally informative for gene-function prediction when tested on the same set of genes.

To further compare the causal expression-based approach with Mashup (in this case including all 18,362 genes covered by Mashup) we determined the top-scoring genes for all three diseases discussed in Section 3.3 with both methods, focusing only on genes that are predicted, i.e., not already associated with the disease in the QKB. Results are presented as heat map plots in Figure S6, showing that top-scoring gene sets computed for both methods are mostly disjoint. For psoriasis a number of top genes obtained with Mashup (CXCR2, MMP9, FLT4, IL12B) are also picked up (with lower scores) by our approach, however some with the opposite sign. There is also some overlap for pulmonary hypertension where the causal approach also gives high scores for Mashup's top scoring genes ACVR2A and IL15. In the network discussions in Section 3.3 (main text) and Section 4 (here) we showed that some of our predicted genes could be verified through an independent literature search (AD: SLITRK5, LOX; PH: AQP11, ACVR2A; psoriasis: BANF1, KLK5, TNIP1). Likewise, some genes predicted by Mashup have been associated with the respective disease (AD: GSAP [17], APH1A [18]; PH: TGFPR1 [19]). By and large both approaches appear to be mostly complementary, highlighting the crucial difference in the underlying information used to encode genes, on one side relationships between indirect causal effects on

expression signatures, and on the other local molecular interactions. This suggests a possible integration of both approaches in future work.

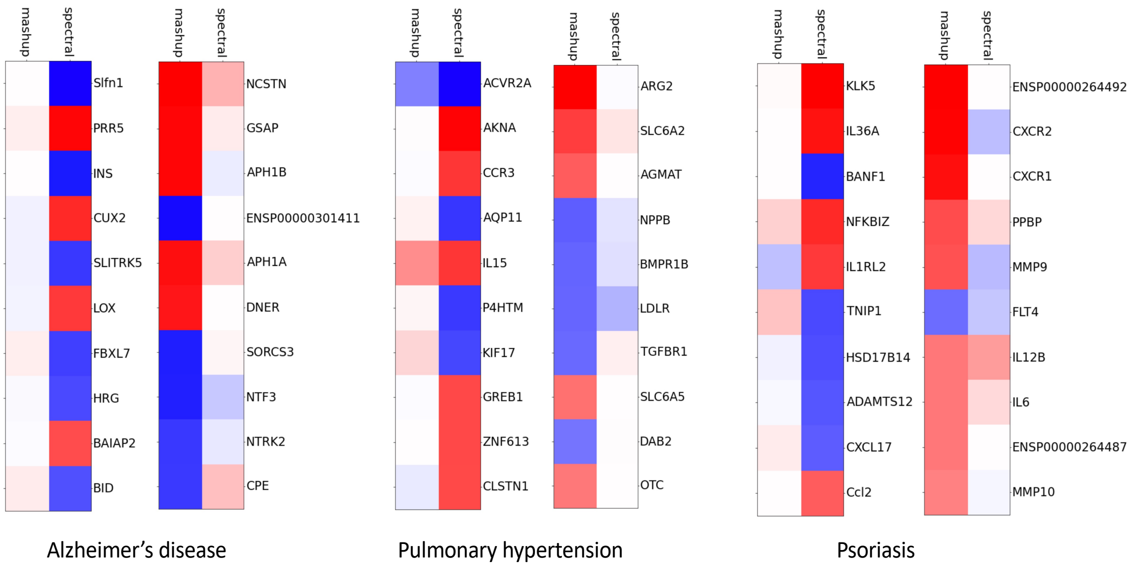

Figure S6. Comparison to Mashup. Top-scoring predicted genes obtained with the spectral model and Mashup for the diseases Alzheimer's disease, pulmonary hypertension, and psoriasis discussed in Section 3.3. Results are presented as heat map plots showing that top-scoring gene sets computed for both methods are mostly disjoint. For psoriasis, several top genes obtained with Mashup (CXCR2, MMP9, FLT4, IL12B) are also picked up (with lower scores) by our approach, however some with the opposite sign. There is also some overlap for pulmonary hypertension where the causal approach also gives high scores for Mashup's top scoring genes ACVR2A and IL15. (Red: activated, blue: inhibited)

7. Supplementary code

The complete code implementing the three embedding models E1, E2, and E3 as well as code needed to perform the analyses described in this paper is provided in two Python Jupyter notebooks. We also provide a subset of the QIAGEN Knowledge Base content as needed to run this code.

7.1. Subset of QKB content

This is provided in the following files:

`expression-bipartite-graph-genes.txt.gz`	Bipartite graph of literature-derived expression regulation patterns of 1500 genes that are most connected
`expression-bipartite-graph-genes+drugs.txt.gz`	Literature-derived expression regulation patterns also including those of the100 most connected drugs
`gene-function-bipartite-graph.txt.gz`	Gene-function/disease bipartite graph containing 1500 genes from above, 400 functions and 100 diseases
`included_drugs.txt`	List of all drugs included above
`included_diseases.txt`	List of all diseases included above
`drug-labels-and-clinical-studies.txt`	Table of drug-disease associations from drug labels and clinical trials including drugs and diseases from above

Entities included in the provided subset of QKB content were selected in the following way:

(1) Genes are the 1500 ones that have the greatest number of connections in the gene-expression bipartite graph.

(2) Drugs are the 100 ones that have the greatest number of connections in the expression bipartite graph and are also part of the list of drugs in the table with drug-disease associations.

(3) To construct the set of functions and diseases included in the test data, we first built a set ("set 1") of all functions and diseases that are regulated by at least 5 genes in the gene-function bipartite graph. Another set ("set 2") was created from the 20 top-scoring functions (as reported in this paper) using the complete model E1 for each of the three diseases discussed in the paper. These three diseases themselves (Psoriasis, Alzheimer's disease, and Pulmonary Hypertension) were also included in "set 2". We then randomly sampled functions and diseases from "set 1" and combined with "set 2" such that the total number of included functions is 400, and the total number of diseases is 100. We also required that each included disease appears in the table with drug-disease associations.

7.2 Jupyter notebooks

We provide two Python Jupyter notebooks, the first one (`embedding_computations.ipynb`) for computing embeddings using the three models, E1, E2, E3, and the second one (`analysis.ipynb`) for running various analyses on the computed embeddings. We also provide precomputed embeddings (using the code in `embedding_computations.ipynb`) in the files `E1_gene_embeddings.txt` (spectral model), `E2_gene_embeddings.txt` (neural network model), and `E3_gene_embeddings.txt` (node2vec), as well as drug embeddings in `E1_drug_embeddings.txt` (for E1) and function/disease embeddings (`[E1|E2|E3]_function_disease_embeddings.txt`).

Supplementary references

[1] Long, Justin M., and David M. Holtzman. 2019. “Alzheimer Disease: An Update on Pathobiology and Treatment Strategies.” Cell 179 (2): 312–39.

[2] De Strooper, Bart, and Eric Karran. 2016. “The Cellular Phase of Alzheimer’s Disease.” Cell 164 (4): 603–15.

[3] Mathys, Hansruedi, Jose Davila-Velderrain, Zhuyu Peng, Fan Gao, Shahin Mohammadi, Jennie Z. Young, Madhvi Menon, et al. 2019. “Single-Cell Transcriptomic Analysis of Alzheimer’s Disease.” Nature 570 (7761): 332–37.

[4] Czapski, Grzegorz A., Kinga Czubowicz, Joanna B. Strosznajder, and Robert P. Strosznajder. 2016. “The Lipoxygenases: Their Regulation and Implication in Alzheimer’s Disease.” Neurochemical Research 41 (1-2): 243–57.

[5] Kuehn, Bridget M. 2020. “In Alzheimer Research, Glucose Metabolism Moves to Center Stage.” JAMA: The Journal of the American Medical Association 323 (4): 297–99.

[6] Wolfe, Devin M., Ju-Hyun Lee, Asok Kumar, Sooyeon Lee, Samantha J. Orenstein, and Ralph A. Nixon. 2013. “Autophagy Failure in Alzheimer’s Disease and the Role of Defective Lysosomal Acidification.” The European Journal of Neuroscience 37 (12): 1949–61.

[7] Song, Minseok, Joanna Giza, Catia C. Proenca, Deqiang Jing, Mark Elliott, Iva Dincheva, Sergey V. Shmelkov, et al. 2015. “Slitrk5 Mediates BDNF-Dependent TrkB Receptor Trafficking and Signaling.” Developmental Cell 33 (6): 690–702.

[8] Sharifi Kia, Danial, Kang Kim, and Marc A. Simon. 2021. “Current Understanding of the Right Ventricle Structure and Function in Pulmonary Arterial Hypertension.” Frontiers in Physiology 12 (May): 641310.

[9] Klouda, Timothy, and Ke Yuan. 2021. “Inflammation in Pulmonary Arterial Hypertension.” Advances in Experimental Medicine and Biology 1303: 351–72.

[10] Li, Min, Suzette Riddle, Sushil Kumar, Joanna Poczobutt, B. Alexandre McKeon, Maria G. Frid, Maureen Ostaff, et al. 2021. “Microenvironmental Regulation of Macrophage Transcriptomic and Metabolomic Profiles in Pulmonary Hypertension.” Frontiers in Immunology 12 (March): 640718.

[11] Karmouty-Quintana, Harry, Kemly Philip, Luis F. Acero, Ning-Yuan Chen, Tingting Weng, Jose G. Molina, Fayong Luo, et al. 2015. “Deletion of ADORA2B from Myeloid Cells Dampens Lung Fibrosis and Pulmonary Hypertension.” FASEB Journal: Official Publication of the Federation of American Societies for Experimental Biology 29 (1): 50–60.

[12] Mertens, Tinne C. J., Ankit Hanmandlu, Ly Tu, Carole Phan, Scott D. Collum, Ning-Yuan Chen, Tingting Weng, et al. 2018. “Switching-Off Adora2b in Vascular Smooth Muscle Cells Halts the Development of Pulmonary Hypertension.” Frontiers in Physiology 9 (June): 555.

[13] International PPH Consortium, K. B. Lane, R. D. Machado, M. W. Pauciulo, J. R. Thomson, J. A. Phillips 3rd, J. E. Loyd, W. C. Nichols, and R. C. Trembath. 2000. “Heterozygous Germline Mutations in BMPR2, Encoding a TGF-Beta Receptor, Cause Familial Primary Pulmonary Hypertension.” Nature Genetics 26 (1): 81–84.

[14] Sharmin, Nahid, Chinyere Chioma Nganwuchu, and Md Talat Nasim. 2021. “Targeting the TGF-β Signaling Pathway for Resolution of Pulmonary Arterial Hypertension.” Trends in Pharmacological Sciences 42 (7): 510–13.

[15] Rabinovitch, Marlene. 2012. “Molecular Pathogenesis of Pulmonary Arterial Hypertension.” The Journal of Clinical Investigation 122 (12): 4306–13.

[16] Pugliese, Steven C., Jens M. Poth, Mehdi A. Fini, Andrea Olschewski, Karim C. El Kasmi, and Kurt R. Stenmark. 2015. “The Role of Inflammation in Hypoxic Pulmonary Hypertension: From Cellular Mechanisms to Clinical Phenotypes.” American Journal of Physiology. Lung Cellular and Molecular Physiology 308 (3): L229–52.

[17] He, Gen, Wenjie Luo, Peng Li, Christine Remmers, William J. Netzer, Joseph Hendrick, Karima Bettayeb, et al. 2010. “Gamma-Secretase Activating Protein Is a Therapeutic Target for Alzheimer’s Disease.” Nature. https://doi.org/10.1038/nature09325.

[18] Schwartzentruber, Jeremy, Sarah Cooper, Jimmy Z. Liu, Inigo Barrio-Hernandez, Erica Bello, Natsuhiko Kumasaka, Adam M. H. Young, et al. 2021. “Genome-Wide Meta-Analysis, Fine-Mapping and Integrative Prioritization Implicate New Alzheimer’s Disease Risk Genes.” Nature Genetics 53 (3): 392–402.

[19] Rol, Nina, Konda Babu Kurakula, Chris Happé, Harm Jan Bogaard, and Marie-José Goumans. 2018. “TGF-β and BMPR2 Signaling in PAH: Two Black Sheep in One Family.” International Journal of Molecular Sciences 19 (9). https://doi.org/10.3390/ijms19092585.

[20] Franceschini, Andrea, Damian Szklarczyk, Sune Frankild, Michael Kuhn, Milan Simonovic, Alexander Roth, Jianyi Lin, et al. 2013. “STRING v9.1: Protein-Protein Interaction Networks, with Increased Coverage and Integration.” Nucleic Acids Research 41 (Database issue): D808–15.

Supplementary Tables

Table S1 (a). Included cell type contexts.

Adhesion vs. Proliferation (see Figure 3a)

mononuclear leukocytes	lymphoid cells
monocytes	neutrophils
dermal cells	lymphocytes
granulocytes	myeloid cells
PBMCs	leukemia cell lines
fibroblast cell lines	bone marrow cell lines
immune cells	lymphatic system cells
keratinocytes	cancer cells
microvascular endothelial cells	endothelial cells
peripheral blood leukocytes	smooth muscle cells
kidney cells	macrophages
epithelial cells	muscle cells
embryonic cells	tumor cells
mast cells	peripheral blood lymphocytes
embryonic cell lines	epithelial cell lines
B lymphocytes	lung cancer cell lines
gonadal cell lines	phagocytes
prostate cancer cell lines	
antigen presenting cells	
red blood cells	
blood cells	
epithelial tissue	
endothelial cell lines	
connective tissue cells	
epidermal cells	

Table S1 (b). Included cell type contexts.

Cell movement vs. Differentiation (see Figure 3b)

lymphoma cell lines	B lymphocytes	bone marrow cells
mononuclear leukocytes	antigen presenting cells	cerebral cortex cells
neuroblastoma cell lines	helper T lymphocytes	cytotoxic T cells
dermal cells	sarcoma cell lines	liver cells
bone marrow-derived macrophages	plasma cells	fibroblasts
peripheral blood monocytes	central nervous system cells	endothelial cells
PBMCs	natural killer cells	effector T lymphocytes
macrophage cancer cell lines	neurons	naive lymphocytes
fibroblast cell lines	thymocytes	astrocytes
brain cells	neural stem cells	monocyte-derived dendritic cells
keratinocytes	vascular smooth muscle cells	carcinoma cell lines
gonadal cells	epidermal cells	osteoclasts
B-lymphocyte derived cell lines	monocyte-derived macrophages	smooth muscle cells
peripheral blood leukocytes	connective tissue cells	hematopoietic cells
memory T lymphocytes	eosinophils	leukemia cells
leukocyte cell lines	neutrophils	tumor cells
epithelial cells	leukemia cell lines	T lymphocytes
embryonic cells	Th2 cells	macrophages
neuroglia	hematopoietic progenitor cells	cancer cells
mast cells	stem cells	muscle cells
nervous tissue cell lines	Schwann cells	epithelial cell lines
embryonic cell lines	breast cell lines	phagocytes
CD4+ T-lymphocytes	heart cells	
neuroblasts	regulatory T lymphocytes	
Th1 cells	tumor cell lines	

Table S2. Alzheimer's disease: top-scoring functions.

function	cosine similarity	function	cosine similarity
Metabolism of acetylcholine	-0.427	Cell death of sensory neurons	0.240
Synaptic transmission of hippocampal neurons	-0.417	Quantity of linoleic acid	-0.237
Quantity of Cajal-Retzius neurons	0.417	Object recognition memory	-0.234
Enlargement of lateral cerebral ventricle	-0.409	Function of brain	-0.229
Removal of Ca2+	0.387	Working memory	-0.228
Acidification of lysosome	0.353	Cell viability of sympathetic neuron	-0.227
Quantity of mesenchymal cells	0.345	Deposition of amyloid fibrils	0.223
Amyloidosis	0.330	Proliferation of adrenal gland cells	-0.221
Activation of spinal neuron	-0.323	Contextual fear memory	-0.219
Cell viability of striatal neurons	-0.305	Quantity of secondary ovarian follicle	-0.217
Inflammation of vessel	-0.302	Synthesis of phosphatidylinositol diphosphate	-0.216
Patterning of embryonic tissue	0.288	Recruitment of microglia	0.216
Synapsis	-0.283	Formation of membrane processes	-0.213
Apoptosis of Schwann cells	-0.282	Extension of neurites	-0.211
Quantity of proteoglycan	0.281	Protection of cortical neurons	-0.211
Aggregation of filaments	0.276	Exocytosis of vesicles	-0.209
Spatial learning	-0.265	Disassembly of microtubules	0.208
Survival of trigeminal ganglion neurons	-0.262	Formation of blastocyst	-0.206
Metabolism of D-glucose	-0.260	Release of L-glutamic acid	-0.203
Chemotaxis of axons	-0.255	Branching of blood vessel	-0.201
Survival of dorsal root ganglion cells	-0.252	Concentration of GABA	-0.201
Activation of ganglion cells	-0.247	Cell movement of astrocytes	-0.201
Density of nerve ending	-0.246	Differentiation of neuroepithelial cells	-0.200
Differentiation of cholinergic neurons	-0.245		
Protection of interneurons	-0.242		

Table S3. Alzheimer's disease: top-scoring genes.

gene	z-score	gene	z-score	gene	z-score	gene	z-score
PSEN1*	13.970	SLC17A6	4.459	YOD1	3.722	HTR1A	3.291
PSEN2*	13.545	NREP	4.438	WNT2B	3.674	CLASP2	-3.279
NGF*	-9.819	CFI	-4.417	FBXO2	3.673	NTF4	-3.268
Slfn1	-7.192	ALB*	-4.387	EIF4E2	3.648	Atg5	3.225
APOE*	7.096	NEUROG2	-4.274	IGSF1	3.641	ZNF76	-3.223
PRR5	7.023	CASP2	-4.161	REST	3.615	ICMT	-3.219
APP*	6.482	MARCHF5	4.063	DAPK3	3.597	AIPL1	-3.211
INS*	-6.423	PLOD1	4.055	CDH5	-3.555	IRF8	-3.201
HMGCR*	6.390	DLX5	-4.052	MAML2	3.441	CYP11B2	-3.198
BDNF*	-6.165	INTS11	-4.051	MAML3	3.441	LRRN1	3.188
CUX2	5.992	SRSF10	4.014	GH2	3.434	AGT	-3.182
SLITRK5	-5.611	DLX6	-4.012	ZSCAN21	3.431	PDCD5	-3.176
LOX	5.509	PHF5A	-3.987	CREM	-3.423	GUCY2F	-3.175
FBXL7	-5.342	CPEB3	-3.972	PAQR3	3.422	EIF2B2	-3.172
HRG	-5.089	GAB1	-3.948	IFT20	3.402	PTGIS	3.165
BAIAP2	5.019	SIM1	-3.920	HCK	-3.390	LLGL2	3.158
BID	-4.931	APPL1	-3.886	ADAM19	-3.386	PIM1	-3.133
PSENEN*	4.894	TTYH1	3.842	NFIC	-3.378	CFB	-3.125
HSPE1	-4.824	SLC8A1	-3.823	MIB1	3.371	BAX	-3.111
SLC30A3	-4.670	SV2A	-3.811	HSP90AA1	3.363	RELN	3.081
Csl	4.592	NEURL1	-3.802	ATP2A1	-3.348	ALOX12	-3.069
B4GALNT1	4.518	CST7	-3.790	PLAU*	-3.342	FZD8	3.068
AMOT	-4.484	ARNT2	-3.760	RHBDF1	-3.327	WNT16	3.062
AMOTL2	-4.484	BATF2	-3.756	SCGB3A2	3.326	PLEKHM1	3.059
AMOTL1	-4.484	HLA-B	-3.745	ATF6	-3.305	NSF	-3.048

Genes marked with (*) are associated with Alzheimer's disease in the QKB. Genes not marked with (*) are predicted to be causally associated.

Table S4. Pulmonary hypertension: top-scoring functions.

function	cosine similarity	function	cosine similarity
Recruitment of eosinophils	0.337	Proliferation of effector memory T lymphocytes	0.210
Muscularization of artery	0.312	Differentiation of naive lymphocytes	0.209
Proliferation of cytotoxic T cells	0.293	Binding of stromal cells	0.208
Secretion of mucus	0.285	Synthesis of leukotriene C4	0.207
Systolic pressure of right ventricle	0.284	Cell viability of monocyte-derived dendritic cells	0.202
Function of helper T lymphocytes	0.272	Remodeling of vascular tissue	0.201
Transmigration of macrophages	0.268	Synthesis of prostaglandin	0.201
Proliferation of invariant natural killer T cells	0.264		
Th2 immune response	0.257		
Generation of bone marrow-derived dendritic cells	0.252		
Stimulation of mast cells	0.252		
Activation of natural killer T lymphocytes	0.250		
Pulmonary fibrosis or aplastic anemia	0.247		
Quantity of adenosine	0.246		
Quantity of Th2 cells	0.245		
Expansion of antigen presenting cells	0.240		
Cell movement of basophils	0.236		
Proliferation of Th1 cells	0.233		
NK cell proliferation	0.233		
Production of superoxide	0.223		
Quantity of phagocytes	0.223		
Development of follicular T helper cells	0.219		
Maturation of natural killer cells	0.218		
Effector phase	0.217		
Stimulation of monocytes	0.216		

Table S5. Pulmonary hypertension: top-scoring genes.

gene	z-score	gene	z-score	gene	z-score	gene	z-score
ADORA2B*	9.536	PAK2	4.334	CEBPZ	3.620	PLAT*	-3.293
ADA*	-7.493	ZNF668	-4.288	NAGLU	3.599	NCR3LG1	3.283
IL18*	7.135	NOS3*	-4.204	NT5E	-3.577	SMAD5	-3.271
PTGDR2*	6.693	LOC290071	-4.184	Havcr1	-3.551	CD82	3.244
ACVR2A	-6.193	JAG2	-4.143	IL3	3.550	CD180	3.237
AKNA	6.056	VIP*	-4.082	HSPB2	-3.547	ACVR1	-3.237
BMPR2*	-6.043	PTGIR	-4.064	MOV10L1	3.542	MXD3	-3.231
RPTOR*	5.773	HJV	-4.024	MS4A1	-3.532	EPAS1*	3.231
APOE*	-4.927	CSF1	4.017	ADORA1	-3.492	NAA30	-3.205
CCR3	4.846	CAV1*	-3.999	GRB10	-3.489	IL33	3.200
AQP11	-4.839	CD83	-3.967	IL17RB	3.483	STOX1	-3.194
IL15	4.838	POU2AF1	3.951	IFITM3	-3.474	TRAF4	3.193
ARG1*	4.813	CCR6	3.930	ALPL	-3.474	ANXA13	-3.178
P4HTM	-4.765	BID	3.918	IL5	3.413	SRFBP1	-3.171
IL1RN*	-4.741	CHRNA1	-3.912	TMPRSS6	3.410	SETDB1	3.165
CSF2*	4.724	FBXO11	-3.905	Til1	3.384	P2RY1	-3.160
EGLN1*	-4.678	BIRC5*	3.896	DAZAP2	-3.373	TNFRSF4	3.119
TNFSF4*	4.636	ARNT	3.863	BLVRA	-3.352	PRMT7	-3.100
KDR*	-4.514	GDF2*	-3.856	CD48	3.351	SPI1	3.069
CXCR4*	4.494	TNFSF10*	3.818	IL2RA	-3.339	CD2	3.050
KIF17	-4.481	ENDOG	-3.765	ICOS	3.331	TFR2	-3.045
GREB1	4.444	Pln	-3.706	WNT2	3.324	CFB	3.036
ZNF613	4.441	Adora3/LOC100911796	3.676	IL9R	3.315	IRF8	3.020
NOS2*	4.438	CAMTA1	3.676	STAT1	3.305	HIF1AN	-3.016
CLSTN1	4.391	ZNF260	3.676	FOSL2*	3.298	USP22	3.013

Genes marked with (*) are associated with pulmonary hypertension in the QKB. Genes not marked with (*) are predicted to be causally associated.

Table S6. Psoriasis: top-scoring functions.

function	cosine similarity	function	cosine similarity	function	cosine similarity	function	cosine similarity
Influx of neutrophils	0.414	Activation of synovial fibroblasts	0.306	Accumulation of pyruvic acid	-0.275	Proliferation of colony-forming granulocyte-macrophages	0.253
Activation of Th17 cells	0.394	Adhesion of mesenchymal stem cells	0.304	Necroptosis of bone marrow-derived macrophages	0.274	I-kappaB kinase/NF-kappaB cascade	0.253
Migration of Langerhans cells	0.393	Synthesis of 5,6,7,8-tetrahydrobiopterin	0.303	Permeability of tight junctions	0.273	Neurogenesis of neural stem cells	0.253
Th17 immune response	0.386	Fever	0.302	Activation of mesangial cells	0.272	Proliferation of fibroblast-like synoviocytes	0.253
Quantity of IL-1a in blood	0.371	Immune response of brain	0.302	Release of L-cysteine	0.272	Generation of Th9 cells	-0.253
Activation of Th1 cells	0.370	Killing of Haemophilus influenzae	0.295	Inflammation of absolute anatomical region	0.272	Binding of Sertoli cells	0.252
Loss of proteoglycan	0.368	Killing of Listeria monocytogenes 10403S	0.295	Binding of E. coli	0.271	Trafficking of B lymphocytes	0.252
Production of anti-DNA antibody	0.357	Polarization of T-cell hybrid cells	0.295	Clearance of Pseudomonas aeruginosa	0.270	Binding of microvessel	0.251
Quantity of nitric oxide	0.354	Release of prostaglandin E2	0.293	Growth of Mycobacterium tuberculosis	-0.269	Acute phase reaction	0.251
Formation of nitrite	0.353	Proinflammatory response	0.293	Dissemination of Klebsiella pneumoniae	-0.269	Efflux of sphingomyelin	-0.250
Apoptosis of thyroid cells	0.337	Fragmentation of DNA fragment	0.292	Formation of PML nuclear bodies	0.269	Activation of alveolar macrophages	0.250
Synthesis of	0.336	Clearance of	0.292	Activation of	0.268	Induction of muscle	0.250

leukotriene B4		Staphylococcus aureus		myeloid-derived suppressor cells		cells	
Translocation of granules	0.335	Cellular infiltration by CD4+ T-lymphocytes	0.292	Induction of follicular T helper cells	0.267	Increased localization of AST	0.249
Stimulation of lung cells	0.335	Stimulation of hepatocytes	0.287	Activation of bone marrow-derived dendritic cells	0.266	Quantity of enterobacteriaceae	-0.249
Cell movement of naive B cells	0.334	Apoptosis of microglia	0.286	Stimulation of chondrocytes	0.264	Apoptosis of trophoblast cells	0.248
Adhesion of peripheral blood monocytes	0.332	Proliferation of endometriotic stromal cells	0.285	Damage of genitourinary system	0.264	Outgrowth of lymph vessel	0.247
Degradation of connective tissue	0.323	Release of sphingolipid	0.284	Release of non-esterified fatty acid	0.263	Apoptosis of effector memory T lymphocytes	0.247
Stimulation of airway smooth muscle cells	0.319	Response of skeletal muscle	0.282	Induction of histamine	0.263	Chemotaxis of Th2 cells	0.246
Scaling of skin	0.317	Permeability of microvasculature	0.280	Activation of sensory neurons	0.261	Maturation of myeloid dendritic cells	0.246
Activation of keratinocytes	0.316	Chemotaxis of basophils	0.280	Release of cyclic GMP	0.260	Binding of C/ebp beta binding site	0.243
Induction of prostaglandin	0.312	Quantity of apoptotic endocrine cell lines	0.280	Writhing	0.256	Apoptosis of islets of Langerhans	0.243
Cell movement of memory B cells	0.310	Response of fibroblasts	0.279	Survival of Francisella tularensis	-0.255	Stimulation of vascular endothelial cells	0.242
Concentration of epoprostenol	0.310	Hematopoiesis of myeloid progenitor cells	-0.279	Activation of natural killer T lymphocytes	0.255	Apoptosis of placenta	0.241
Inflammatory infiltrate	0.307	Release of reactive oxygen species	0.278	Killing of Leishmania	0.254	Chemoattraction of eosinophils	0.241
Cell viability of oligodendrocyte precursor cells	-0.307	Stimulation of hyaluronic acid	0.277	Binding of vascular smooth muscle cells	0.253	Flux of Ca2+	0.240

Table S7. Psoriasis: top-scoring genes.

gene	z-score	gene	z-score	gene	z-score	gene	z-score
KLK5	9.014	GABRA2	5.358	FILIP1	-3.917	STAT1	3.634
DTL*	-8.339	ACKR2*	-5.264	SIGIRR*	-3.891	CCL20*	3.609
IL36A*	8.312	IL1B	5.150	ARRB1	3.884	S100A4	3.607
IL17C*	8.289	IL10*	-5.130	TGFB1*	3.880	AGER	3.603
TEK*	7.871	IL22RA2*	-5.038	IL12RB2	-3.862	IFNL1	3.564
BANF1	-7.736	IL22*	4.824	PRKCA	3.831	CCR2	3.558
NFKBIZ	7.492	TCL1A	4.761	PLAUR	3.823	TNFSF12	3.547
PLA2G2D*	-7.490	IL4*	-4.713	S100A8	3.822	IFNL3	3.513

IL17A*	7.329	CNPY3	4.676	Tcrd	3.818	CYFIP2	-3.500
IL23A*	7.180	CXCL8*	4.535	Rcan1	3.818	NPC1	-3.493
IL1RL2	6.931	CFP	4.534	PNPT1	-3.789	CD200	-3.486
TNF*	6.862	IRF7	4.482	IL1A	3.771	IL12B	3.481
TNIP1	-6.362	CXCR5	4.471	IL20*	3.754	PF4	3.470
HSD17B14	-6.263	SCGB1A1	-4.411	SWAP70	3.751	TNFAIP3	-3.445
IL1RN*	-6.240	NPS	4.385	IL1RL1	-3.740	TRHR	3.445
ADAMTS12	-5.942	Wfdc17	-4.383	CSF3R	-3.738	GLIS1	3.415
CXCL5*	5.931	TRAIP	-4.364	IL36G	3.726	HSP90B1	3.414
CXCL17	-5.741	ZFP36	-4.339	CXCL1*	3.718	RBBP4	3.402
Ccl2*	5.738	TRU-TCA1-1	-4.261	OPA1	-3.712	Usp17la	3.392
CAMP	5.652	CCR1	4.205	C7	3.669	RGS10	-3.375
KDR	-5.582	IRF3	4.113	STAT6	-3.663	ZBTB46	-3.373
GPR34	5.424	PTPN22	-4.100	PTPRT	-3.653	TRAF3IP2*	3.372
IFNG	5.396	CEBPE	4.048	Saa3	3.650	IL23R	3.333
Ctf2	5.393	CXCL2	3.965	APOA1	-3.646	ANKRD17	3.330
REG3A	5.393	MFAP2	-3.941	APP	3.643	PGRMC1	3.318

Genes marked with (*) are associated with psoriasis in the QKB. Genes not marked with (*) are predicted to be causally associated.
